# The gut microbiota in high-altitude medicine: intersection of hypoxic adaptation and disease management

**DOI:** 10.3389/fmicb.2025.1705487

**Published:** 2025-11-05

**Authors:** Qian Chen, Demei Huang, Junling Liu, Nan Jia, Zherui Shen, Caixia Pei, Chen Chen, Yuhan Liu, Yilan Wang, Shihua Shi, Renxing Yi, Yacong He, Fei Wang, Zhenxing Wang

**Affiliations:** ^1^Hospital of Chengdu University of Traditional Chinese Medicine, Chengdu, China; ^2^Friedrich Miescher Institute for Biomedical Research (FMI), Basel, Switzerland; ^3^Department of Pediatrics, Harvard Medical School, Boston Children's Hospital, Boston, MA, United States; ^4^School of Pharmacy, Chengdu University of Traditional Chinese Medicine, Chengdu, China

**Keywords:** gut microbiota, hypoxic adaptation, altitude-related disorders, microbiome-host crosstalk, microbiome-targeted interventions

## Abstract

High-altitude exposure impacts hundreds of millions globally, posing a unique health challenge due to extreme stressors including hypobaric hypoxia and intense ultraviolet radiation. The gut microbiota, a microbial community residing in the intestinal tract, plays a pivotal role in maintaining host health through homeostasis. Emerging evidence highlights the gut microbiome's dual roles in facilitating host adaptation to high-altitude environments and in mediating maladaptive responses. This review explores the potential changes and mechanisms of the gut microbiota and its metabolites in mediating host adaptation and pathogenesis related to high-altitude exposure, alongside summarizing effective strategies for targeted microbiota modulation to prevent and treat altitude-related disorders. Furthermore, we discuss the influence of microbiota on drug metabolism in high-altitude populations and its potential role as diagnostic and prognostic biomarkers. Although current research remains exploratory, the gut microbiome has garnered significant interest in high-altitude medicine. With advancing investigations, microbiota-targeted interventions may emerge as critical breakthroughs for altitude disease management, paving the way for improved human adaptation to extreme environments and precision health strategies for plateau populations.

## 1 Introduction

High-altitude environments, characterized by extreme stressors such as hypobaric hypoxia, low temperatures, and intense ultraviolet radiation, pose significant challenges to human health worldwide. Over 80 million people live permanently above 2,500 m, with millions more temporarily exposed through travel, occupational engagements, or military deployments (Gatterer et al., 2024). Ecogeographically distinctive regions including the Qinghai-Tibetan Plateau, Andean Highlands, and Rocky Mountains have become focal areas for altitude-associated health research. Epidemiological studies demonstrate that 35–73.5% of non-acclimatized individuals develop acute mountain sickness (AMS) when exposed to altitudes exceeding 3,000 m (Cobb et al., 2021; Croughs et al., 2014), presenting with headache, nausea, and fatigue. Severe manifestations may escalate to life-threatening high-altitude pulmonary edema (HAPE) and cerebral edema (HACE; Imray et al., 2011). Epidemiological studies report that the incidence of HAPE and HACE varies considerably worldwide, ranging from less than 0.01% to 31% (Basnyat et al., 2000; Korzeniewski et al., 2015; Savioli et al., 2022). Prolonged residency at high elevations correlates with increased susceptibility to pathological conditions such as chronic mountain sickness (excessive erythrocytosis), pulmonary hypertension, high-altitude heart disease, cognitive deficits, and metabolic dysfunction (Richalet et al., 2024; Zhang et al., 2022; Zila-Velasque et al., 2024). Emerging researches emphasize the complex interactions between environmental stressors and systemic physiological adaptations, by focusing on oxidative stress/antioxidant defense mechanisms, mitochondrial energy metabolism, immune system modulation, and neuroendocrine equilibrium. These pathways collectively represent critical frontiers in understanding altitude adaptation pathophysiology (Pena et al., 2022).

The physiological challenges of high-altitude exposure originate from systemic disturbances triggered by hypobaric hypoxia, which subsequently drive multiorgan dysfunction and disease (Imray et al., 2010). At the cellular level, hypoxia disrupts mitochondrial energy metabolism by shifting ATP production from oxidative phosphorylation to anaerobic glycolysis—a transition mediated by hypoxia-inducible factor-1α (HIF-1α; Papandreou et al., 2006). While this adaptive mechanism conserves oxygen, it exacerbates oxidative injury through accelerated reactive oxygen species accumulation due to electron transport chain leakage, overwhelming the capacity of endogenous antioxidants such as superoxide dismutase and glutathione peroxidase (Hernansanz-Agustin et al., 2017). Concurrently, hypoxia-driven sympathetic nervous system activation and hyperactivity of the hypothalamic-pituitary-adrenal axis contribute to heightened cardiovascular strain and reduced metabolic efficiency (Krugers et al., 1995; Simpson et al., 2024). These effects are further compounded by immune dysregulation: hypoxia-induced proinflammatory cytokines [e.g., Interleukin (IL)-6, tumor necrosis factor (TNF)-α] disrupt the T helper cell 17/Regulatory T cells balance, aggravating endothelial damage and vascular leakage in HAPE (Ulloa and Cook, 2025). These interconnected mechanisms—mitochondrial metabolic reprogramming, oxidative stress, neuroendocrine hyperactivity, and immune-inflammatory crosstalk—collectively form the pathological network underlying altitude-related disorders. Notably, emerging evidence suggests that gut microbiota may modulate these pathways via microbial metabolites, immune priming, and neuroendocrine interactions (Gao et al., 2020; Kim, 2023), positioning host-microbe dialogue as a critical yet underexplored axis in high-altitude medicine.

The gut microbiota, a symbiotic microbial community residing in the host gastrointestinal tract, serves as a dynamic interface between environmental stressors and host physiology. Through metabolic, immune, and neuroendocrine interactions, gut microbes influence energy harvesting, inflammatory responses, and stress resilience—processes critical for high-altitude adaptation (Pennisi, 2024). Intriguingly, high-altitude natives in regions like the Himalayas and the Andes possess unique gut microbial profiles characterized by enrichment of fiber-fermenting taxa and short-chain fatty acids (SCFAs)-producing bacteria, which are hypothesized to contribute to enhanced hypoxic tolerance (Quagliariello et al., 2019). Conversely, lowland migrants who experience acute mountain sickness frequently demonstrate gut dysbiosis characterized by reduced microbial diversity and proliferation of opportunistic pathogens (Han et al., 2021). Emerging research observations position the gut microbiota as both a biomarker and a critical regulator of host health and altitude adaptation. Accordingly, this review aims to synthesize the current evidence regarding changes in the gut microbiota and their functional metabolites in response to high-altitude exposure. Our approach involves a comprehensive integration of key findings from human studies, animal models, and intervention trials. We focus on elucidating how these microbial alterations may mediate physiological adaptation and contribute to the pathogenesis of altitude-related illnesses. Furthermore, we explore the translational implications of these findings by highlighting novel perspectives and emerging therapeutic strategies targeting the gut microbiota for improved management of high-altitude health.

## 2 Gut microbiota

### 2.1 Composition and function of gut microbiota

The gut microbiota refers to the microbial communities residing in the human gastrointestinal tract, primarily composed of bacteria, archaea, fungi, and viruses, with bacteria being the main focus of research. At the phylum level, *Firmicutes* and *Bacteroidetes* represent the dominant groups, collectively accounting for 70% to 90% of the total bacterial population, and their relative abundance ratio is a classical indicator of microbial homeostasis (Adak and Khan, 2019). Among the subdominant phyla, *Bifidobacterium* from *Actinobacteria* acts as a typical probiotic by inhibiting pathogen growth through acid production; *Proteobacteria* includes opportunistic pathogens such as *Escherichia coli*, whose elevated abundance is often associated with impaired intestinal barrier function; and *Akkermansia muciniphila* from *Verrucomicrobia* participates in barrier repair by degrading the intestinal mucus layer and is considered a key indicator of gut health (Adak and Khan, 2019; Jandhyala et al., 2015). Through diverse metabolic functions, the gut microbiota modulates host energy metabolism, immune responses, and signaling pathways. Important categories of microbiota-derived metabolites include SCFAs, bile acids, amino acids, trimethylamine N-oxide (TMAO), tryptophan, and indole derivatives (Agus et al., 2021). SCFAs, such as acetate, propionate, and butyrate, are major metabolites derived from microbial fermentation of dietary fiber (Koh et al., 2016). They serve not only as energy sources but also modulate inflammatory responses and enhance intestinal barrier integrity through interactions with host cell receptors (He et al., 2020). Bile acids play key roles in lipid metabolism and immune regulation (Lin et al., 2023). Tryptophan and its metabolites, including serotonin and indole compounds, are critically involved in neurological functions, influencing host mood and behavior (Gao et al., 2020). TMAO contributes to cardiovascular disease pathogenesis by modulating endothelial function (Witkowski et al., 2020). As a highly diverse and dynamic ecosystem, the structural stability and functional integrity of the gut microbiota are essential for host resistance to environmental stressors and the maintenance of physiological homeostasis.

### 2.2 Impact of high-altitude environments on gut microbiota composition

**Figure 1:**
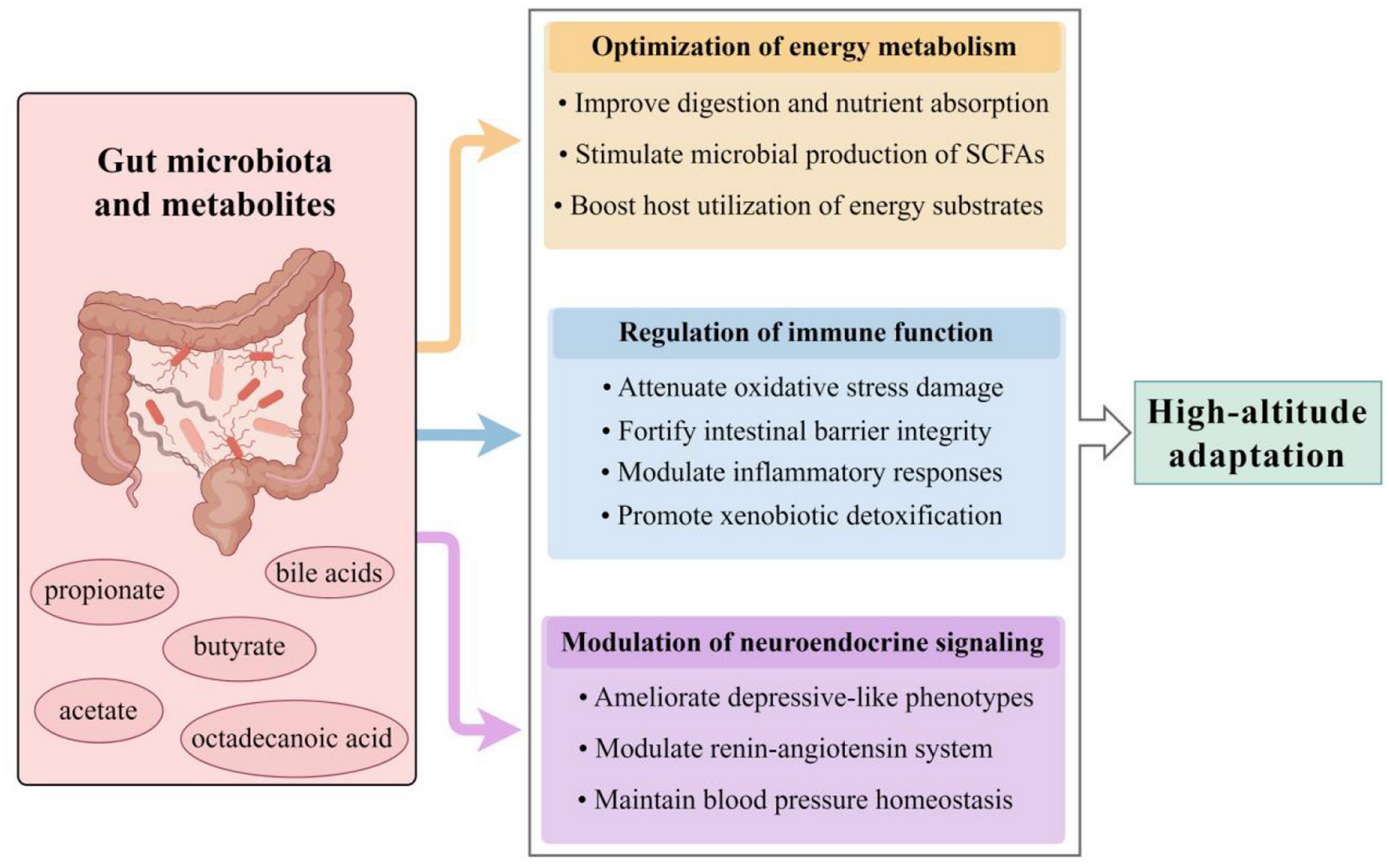
Gut microbiota-mediated mechanisms of high-altitude adaptation. The gut microbiota and its metabolites facilitate host acclimatization through three synergistic pathways. Microbial remodeling enhances systemic energy availability through improved dietary energy harvest and production of key metabolites such as SCFAs. Simultaneously, immune homeostasis is maintained by alleviating oxidative stress and inflammation, strengthening the intestinal barrier, and promoting xenobiotic detoxification. Additionally, microbiota-derived neurotransmitters and metabolites modulate neuroendocrine signaling, contributing to the regulation of mood and blood pressure. SCFAs, short-chain fatty acids (by Figdraw ID: STISIa9fa4.).

The unique environmental stressors at high altitude—hypoxia, cold, and dietary constraints—drive a remodeling of the gut microbial community in both structure and function. For instance, Tibetan highlanders exhibit gut microbiota enriched with *Prevotella* and *Bifidobacterium*, taxa associated with enhanced polysaccharide fermentation capacity and anti-inflammatory SCFAs production, mediating host adaptation to plateau hypoxia and reduced disease susceptibility (Liu et al., 2021). Parallel studies reveal elevated abundances of the butyrate-producing *Faecalibacterium* in Qinghai-Tibetan Plateau populations, correlating with intestinal barrier integrity and mitigation of chronic mountain sickness (CMS)-associated inflammation (Zhao et al., 2024). High-altitude animals such as yaks and Tibetan antelopes harbor *Firmicutes*-predominant microbiomes that optimize energy extraction from fibrous diets—a critical survival trait in resource-scarce alpine ecosystems (Ma et al., 2019; Zhu et al., 2024). Crucially, dysbiosis in these altitude-adapted microbial communities is closely associated with disease predisposition. Lowland migrants experiencing AMS typically demonstrate reduced microbial diversity, *Enterobacteriaceae* overgrowth, and diminished SCFAs levels—alterations linked to increased intestinal permeability and systemic inflammation (McKenna et al., 2022). These observations suggest that the gut microbiota is highly plastic in responding to environmental stressors, highlighting its potential dual role in high-altitude adaptation—where it may act as both a target and a driver of adaptive processes. The subsequent sections will delve deeper into these alterations and their underlying response mechanisms (Figure 1).

## 3 Regulatory functions and mechanisms of gut microbiota in high-altitude adaptation

The gut microbial community exerts a profound influence on various aspects of host health and demonstrates the capacity to adapt to extreme environments and dietary fluctuations (Lee et al., 2025). In recent years, an increasing body of research has highlighted the pivotal role of gut microbiota and their metabolites in facilitating host adaptation to high-altitude environments (Su et al., 2024b; Zhao et al., 2024; Zhou et al., 2025). These microorganisms not only contribute to the digestion and absorption of nutrients, but also regulate key physiological functions, including endocrine metabolism, immune responses, circulation, and nervous system activities (Du et al., 2022; Mazel, 2019; Qiao et al., 2025; Wang L. et al., 2023; Zhu et al., 2020). To provide a comprehensive overview of the current research landscape, this chapter will explore the alterations, functions, and regulatory mechanisms of gut microbiota and their metabolites in the acclimatization to high-altitude conditions across different species.

### 3.1 Optimization of energy metabolism

Current studies have demonstrated that gut microbiota play a pivotal role in high-altitude acclimatization by regulating host energy metabolism (Ma et al., 2025). In fact, a diverse range of species across the globe utilize distinct gut microbial community structures to support their survival and functional activities within specific geographic regions (Arumugam et al., 2011). In the low-oxygen environment of high-altitude regions, the host's energy metabolism undergoes significant adaptations to meet survival demands. The gut microbiota are integral to regulating food digestion and nutrient absorption, influencing various metabolic processes such as glucose, lipid, and protein metabolism.

#### 3.1.1 Animals studies

Studies have shown that Tibetan chickens, compared to lowland chickens, exhibit significantly less microbial diversity at lower altitudes. Upon high-altitude exposure, Tibetan chickens display higher abundances of *Firmicutes, Bacteroidetes, Actinobacteria*, and *Proteobacteria* in their gut microbiota, with a notable increase in *Firmicutes* abundance in the ileum (Du et al., 2022). This microbial shift may promote dietary fermentation and enhance the efficient degradation of carbohydrates and proteins (Waite and Taylor, 2014). Further analysis at the genus level revealed increased abundances of *Bacteroides, Peptoclostridium, Ruminococcus, Bifidobacteriaceae* and *Lactobacillus*. *Bacteroides* are highly adaptable, particularly under hypoxic conditions, due to their possession of a unique multifunctional metabolic pathway (Shin et al., 2024). These bacteria can sense specific polysaccharide degradation intermediates and regulate the transcription of corresponding gene clusters—known as polysaccharide utilization loci—to metabolize the necessary enzymes for degrading a wide range of polysaccharides, including the complex plant polysaccharides such as starch, cellulose, xylans, and pectin (Wexler and Goodman, 2017). This allows *Bacteroides* to swiftly adapt to the nutrient conditions in the gut. Additionally, *Bacteroides* participate in the metabolism of animal proteins, various amino acids, and saturated fatty acids (Ma et al., 2024). Through this dominant metabolic mechanism, *Bacteroides* emerge as key species within the microbial community that influence host metabolism, playing a critical role in high-altitude adaptation. *Bifidobacterium* and *Lactobacillus* are recognized probiotics that regulate gastrointestinal function and enhance alpha- and beta-galactosidase activity (Yuan et al., 2024). Notably, the energy conversion and glycan biosynthesis functions in high-altitude chickens were significantly elevated compared to those in low-altitude chickens. Experimental results from Huang et al. (2022) showed that 4 weeks of hypoxia-induced gut microbiota remodeling in a mouse model led to an increased abundance of *Akkermansia* and *Bacteroides*, as well as their associated SCFAs. Importantly, both acetate and butyrate stimulated mitochondrial synthesis and improved biochemical markers related to exercise fatigue, including blood urea nitrogen, creatine kinase, and lactate levels, ultimately enhancing exercise endurance under hypoxic conditions. However, the specific mechanisms involved in these improvements require further detailed investigation. Similarly, *Ruminococcus, Oscillospira*, and *Clostridium*, both members of the *Firmicutes* phylum, were more enriched in Tibetan antelopes at high altitudes than in low-altitude sheep (Ma et al., 2019). These microbes play a crucial role in promoting carbohydrate fermentation in the gut. Furthermore, functional genes related to carbohydrate and energy metabolism, as well as genes involved in DNA replication, recombination, and repair, were significantly enriched in the gut microbiota of high-altitude antelopes. This suggests a potential co-evolution between the gut microbiota and the host genome during high-altitude acclimatization in antelopes (Ma et al., 2019). However, the specific molecular mechanisms underlying this symbiotic evolution require further investigation and elucidation.

Further studies have demonstrated that the *Firmicutes*-to-*Bacteroides* ratio (F/B ratio) of the gut microbiota is positively correlated with the efficient absorption of dietary energy. *Firmicutes* are believed to encode enzymes related to energy metabolism, producing various digestive enzymes that break down diverse substances, whereas *Bacteroides* primarily degrade carbohydrates and proteins. Notably, in high-altitude mammals, the increased F/B ratio is associated with improved energy utilization from dietary fiber in the gut, as well as enhanced host resistance to cold stress. In rhesus monkeys, which share a high genomic similarity with humans (92–95%), the gut microbial community at high altitudes is dominated by *Firmicutes* and *Ruminococcus*, suggesting that populations living at high altitudes have a greater capacity for energy harvesting and consumption (Wu et al., 2020). Additional functional gene enrichment analysis revealed that, at high altitudes, gut microbiota exhibit an increased potential for utilizing CO_2_ and oxaloacetate in the synthesis of energy substrates such as acetyl coenzyme A and pyruvate, respectively, and in the conversion of acetyl coenzyme A into acetate (Zhao et al., 2023b). This suggests that gut microbiota contribute to high-altitude adaptation in rhesus monkeys through critical energy compensation mechanisms. Furthermore, methanol can serve as a substrate for the microbial production of methane and acetate (Oliphant and Allen-Vercoe, 2019). Correspondingly, the abundance of genes related to methanol metabolism was significantly higher in the gut microbiota of high-altitude populations compared to those from lower altitudes, indicating that gut microbiota in high-altitude populations optimize the utilization of metabolites more efficiently. In Tibetan pigs exposed to high-altitude conditions, the significantly increased relative abundance of certain gut bacterial groups is linked to the sustained enrichment of genes involved in energy, amino acid, and carbohydrate metabolism pathways. Specifically, upregulation of microbial genes leads to increased biosynthesis of propanoic acid and octadecanoic acid in fecal metabolites (Zeng et al., 2020), which is closely associated with host adaptation to high-altitude environments. Studies on aquatic organisms have yielded similar results; the F/B ratio in Nile tilapia at high altitudes was significantly higher than in low-altitude populations, facilitating more efficient energy extraction from dietary nutrients (Bereded et al., 2022).

#### 3.1.2 Human studies

In humans, *Firmicutes, Bacteroidetes, Proteobacteria*, and *Actinobacteria* are the four dominant phyla of the gut microbiota. However, in high-altitude populations, there is a significant enrichment of *Bacteroidetes*, while the relative abundance of *Proteobacteria, Actinobacteria, Fusobacteria*, and other phyla is markedly reduced. Notably, *Faecalibacterium, Dorea, Roseburia*, and *Prevotella_9* are significantly elevated in both Tibetan and Han Chinese populations residing at high altitudes (Zhao et al., 2024). As a result, gut microbiota biomarkers for high-altitude populations are predominantly members of the *Ruminococcaceae* and *Trichosporonaceae* families. Notably, *Lachnospiraceae* and *Ruminococcaceae* are probiotic families known for their production of SCFAs. SCFAs are key metabolic products of the gut microbiota, primarily generated through the glycolytic fermentation of dietary carbohydrates and absorbed in the intestines. They serve as a major energy source for intestinal epithelial cells. Studies have shown that SCFAs not only regulate energy homeostasis by influencing insulin sensitivity and glucose utilization in the host (Ingerslev et al., 2014), but also impact the function of intestinal mucosal cells. This dual role enhances energy intake efficiency in high-altitude populations, aiding in their adaptation to the environment. The main types of SCFAs include succinate, propionate, acetate, and butyrate. Among these, butyrate is the principal energy source for colonic epithelial cells, whereas acetate and propionate are essential for hepatic lipogenesis and gluconeogenesis (Rios-Covian et al., 2017). *Prevotella* is also recognized as a significant SCFAs producer, contributing to a wide range of carbohydrate and protein fermentations critical for maintaining gut homeostasis at high altitudes (Macfarlane and Macfarlane, 2012). Certain *Prevotella* species have been shown to improve glucose homeostasis by increasing glycogen stores (Kovatcheva-Datchary et al., 2015), which may help mitigate impairments in glucose tolerance caused by acute altitude exposure (Young et al., 2018). Nevertheless, *Prevotella* may exhibit deleterious effects under specific environmental conditions or individual differences. For instance, during oxidative stress, certain *Prevotella* species can promote intestinal mucus barrier dysfunction and inflammation (Tett et al., 2021), and some, such as melanin-producing *Prevotella*, may act as opportunistic pathogens. Future research should focus on unraveling the functional diversity of *Prevotella* across different environments and its interactions with the host to further explore its biomedical potential.

A study comparing the gut microbiota of hypertensive patients and healthy individuals at different altitudes revealed that both high-altitude Tibetans and middle-altitude Han Chinese exhibited greater α-diversity (intra-individual microbial diversity) in their gut microbiota compared to those at lower altitudes. Additionally, hypertensive patients exposed to high altitudes demonstrated increased β-diversity (inter-individual microbial compositional variation) and a higher abundance of *Verrucomicrobia* and *Akkermansia* (Zhu et al., 2020). *Verrucomicrobia*, a common phylum within the human gut microbiota, plays a role in host energy metabolism by degrading polysaccharides from intestinal fermentation (Fujio-Vejar et al., 2017). Meanwhile, mucin-degrading *Akkermansia* has been shown to alleviate systemic inflammation and reduce hypertension, which will be discussed in more detail below. Therefore, compared to healthy individuals of the same altitude and ethnicity, the increased abundance of *Verrucomicrobia* and *Akkermansia* may offer significant benefits to hypertensive patients in adapting to high-altitude environments by regulating energy metabolism and modulating inflammatory responses.

Emerging evidence highlights the gut microbiota's capacity to orchestrate host purine-uric acid metabolic crosstalk as a novel axis of high-altitude adaptation. Longitudinal multi-omics profiling of high-altitude migrants reveals hypoxia-driven enrichment of *Escherichia coli* and *Klebsiella pneumoniae* strains harboring upregulated purF (amidophosphoribosyltransferase) expression while downregulated xdh (xanthine dehydrogenase). This microbial shift enhances biosynthesis of purine precursors such as inosine while suppressing uric acid production (Han et al., 2024). Integrated gut metagenomic data from Su et al. (2024a) revealed that residents adapted to high altitude exhibit significantly reduced intestinal uric acid levels, correlating with persistently high abundances of the genera *Collinsella, Blautia A*, and *Enterocloster*—key bacterial taxa involved in uric acid degradation. Such coordinated microbiome remodeling may represent a protective adaptive strategy against hyperuricemia and gout in highland inhabitants.

Collectively, the findings discussed above converge to suggest that remodeling of the gut microbiota under high-altitude stress favors a functional paradigm oriented toward enhanced energy harvest. The consistent enrichment of bacterial taxa such as *Bacteroides, Ruminococcus*, and *Akkermansia muciniphila*, alongside a significant increase in the F/B ratio across multiple species, points to a conserved microbial strategy for optimizing dietary energy extraction. This is functionally supported by increased production of microbial metabolites like acetate and butyrate, which serve as key energy substrates for the host. However, while these structural and metabolic shifts are compelling, they primarily reveal a capacity for enhanced energy harvest. The pivotal question of whether this microbial reprogramming is a primary driver of host energy homeostasis or a secondary consequence of hypoxic stress remains largely unanswered, underscoring the need for future studies capable of experimentally manipulating these microbial functions to establish causality.

### 3.2 Regulation of immune function

Exposure to hypoxia at high altitude disrupts systemic redox homeostasis and induces oxidative stress, which in turn damages the integrity of the intestinal barrier (McKenna et al., 2022). The intestinal barrier serves as a crucial defense between the gut and the internal environment, consisting of mechanical, chemical, immune, and biological components (Zheng et al., 2023). The primary mechanical barrier is formed by intestinal mucosal epithelial cells and tight junction proteins, while the chemical barrier includes mucus secreted by epithelial cells and antimicrobial substances produced by the gut microbiota, effectively preventing the invasion of bacteria and toxins, thereby mitigating severe inflammatory responses and endotoxemia (Chelakkot et al., 2018). The immune barrier is composed of gut-associated lymphoid tissue and secretory antibodies produced by plasma cells, which mediate both humoral and cellular immunity (Neurath et al., 2025). The biological barrier is primarily established by the normal microbiota colonizing the gut. These barriers work synergistically to regulate intestinal immunity and maintain overall health. However, in the high-altitude environment, characterized by low atmospheric pressure and hypoxia, intestinal tissues are vulnerable to hypoxia, ischemia, and acidosis, which compromise the integrity and permeability of the intestinal mucosal barrier. This may result in bacterial translocation, uncontrolled invasion by pathogenic microorganisms, and the onset of local or even systemic inflammatory responses (McKenna et al., 2022). The gut microbiota and its metabolites play a pivotal role in high-altitude adaptation by modulating the host immune system through multiple mechanisms (Ghosh et al., 2021).

#### 3.2.1 Mitigation of oxidative stress

Certain gut microbiota and their metabolites enhance the defense of the gut mucosal barrier by alleviating excessive oxidative stress. Previous studies have reported that members of *Trichosporonaceae* and *Ruminalicoccaceae* exhibit strong antioxidant capacities. The increased abundance of these genera in individuals exposed to high-altitude conditions helps to control oxidative stress and maintain the integrity of the intestinal mucosal barrier (Uchiyama et al., 2022), thereby preventing common gastrointestinal disorders, such as inflammatory bowel diseases, ulcers, and irritable bowel syndrome in high-altitude regions. These bacteria also effectively mitigate the symptoms of high-altitude gastrointestinal conditions, including anorexia, dyspepsia, bloating, epigastric discomfort, and diarrhea. Furthermore, cellular experiments in a high-altitude rat model have shown that butyrate can downregulate the expression of HIF-1α by reducing the activity of lactate dehydrogenase A, which in turn attenuates the glycolytic pathway and alleviates cellular responses to hypoxic stress (Zhao et al., 2024). This mechanism may reduce the extent of intestinal injury induced by high-altitude exposure. These findings suggest that *Trichosporonaceae* and *Ruminalicoccaceae* may resist oxidative stress through an intrinsic mechanism, protecting the host mucosal barrier.

#### 3.2.2 Preservation of gut barrier integrity

Gut microbiota can influence the tight junctions between intestinal epithelial cells, regulate the expression and function of gap junction proteins, and maintain the integrity and selective permeability of the mucosal barrier. Andrani et al. (2024) demonstrated that the gut microbiota metabolites propionate and butyrate attenuate the downregulation of duodenal occludin expression induced by environmental stressors, and that butyrate significantly increases occludin protein expression in hypoxic colonic cells, thereby preserving the integrity of the intestinal barrier. In the previously mentioned study characterizing the gut microbiota of hypertensive patients at high altitude (Zhu et al., 2020), the significantly enriched mucinophilic genus *Akkermansia*, a representative of the *Verrucomicrobia* phylum, was negatively associated with cardiovascular disease and inflammatory response. It has been shown that *Akkermansia* protects the intestinal barrier by increasing the expression of occludin and zona occludens protein 1, thereby preventing lipopolysaccharide translocation, reducing metabolic endotoxemia-induced inflammation, and attenuating atherosclerotic lesions (Li et al., 2016). Furthermore, many studies have consistently demonstrated a positive correlation between hypertension and systemic inflammation (Norlander et al., 2018). In hypertensive conditions, mechanical stretch of varying intensities stimulates the release of pro-inflammatory cytokines from vascular endothelial cells and activates peripheral lymphocytes (Jufri et al., 2015), contributing to the pathological processes of various cardiovascular diseases, including hypertension. Therefore, gut microbiota that exert protective effects on the intestinal mucosal barrier are crucial for human adaptation to hypertension and provide adaptive protection for the cardiovascular system in high-altitude environments. Additionally, in the native high-altitude aquatic species *Gymnocypris przewalskii*, the gut microbiota exhibits a complex composition (Wang et al., 2021). While the genus *Aeromonas* includes recognized fish pathogens, the co-dominant genera *Clostridium* and *Cetobacterium* are associated with beneficial functions, including enhanced intestinal barrier integrity and suppression of inflammation, which may collectively support host survival in the extreme aquatic environment (Guo et al., 2020; Qi et al., 2023; Wang et al., 2021).

#### 3.2.3 Modulation of inflammatory homeostasis

Another crucial immunomodulatory pathway involves the ability of certain intestinal bacterial species, which are enriched in high-altitude regions, to modulate immune cell activity (e.g., dendritic cells, macrophages, T cells). These bacteria promote the proliferation and activation of anti-inflammatory cells, upregulate anti-inflammatory factors, and significantly downregulate pro-inflammatory factors. This modulation effectively inhibits the release of inflammatory mediators and helps to maintain the balance of the local immune microenvironment (Wang L. et al., 2023). For instance, *Bifidobacterium bifidum*, a potent immunomodulator in human diseases, induces the production of regulatory T cells (Verma et al., 2018), regulates helper T cell 2 and Th17-mediated immune responses in the intestine, inhibits the production of pro-inflammatory cytokines such as TNF-α, IL-6, IL-1β, IL-18, IL-22, and IL-9, while promots the expression of anti-inflammatory cytokines such as IL-10, IL-4, and IL-5 (Fan et al., 2021). Numerous species exhibit a significant increase in the abundance of Firmicutes during adaptation to high-altitude exposure, a bacterial phylum that serves as the primary producer of butyrate in the human gut. Notably, key butyrate-producing taxa within this phylum—including *Faecalibacterium prausnitzii, Clostridium, Eubacterium rectale*, and *Roseburia*—demonstrate pronounced enrichment under these conditions. Butyrate, a short-chain fatty acid with anti-inflammatory properties, plays a critical role in immune regulation across various disease models (Nie et al., 2021). In intestinal cells, butyrate inhibits histone deacetylase, thereby activating transcription factors such as signal transducer and activator of transcription 3, transcription-specific protein 1, HIF-1, and nuclear factor kappa-B. This leads to increased production of anti-inflammatory mediators (e.g., IL-10) and antimicrobial peptides, ultimately reducing inflammation (Venegas et al., 2019). Li et al. (2019) reported that *Bacillus subtilis* BS1, BS2 and *Bacillus velezensis* BV1, isolated from a Tibetan yak model, were associated with the downregulation of pro-inflammatory factors TNF-α, IL-6, and IL-8, and the upregulation of anti-inflammatory factor IL-10 and serum antibodies IgG, IgM, and IgA. These effects contribute to the modulation of the inflammatory response and immune status, and may be linked to the butyrate produced by *Bacillus sphaericus*. Additionally, multiple studies have identified *Staphylococcus aureus* as a common opportunistic pathogen in the gut, closely associated with intestinal infections. Hypoxic environments at high altitudes can lead to a significant increase in the abundance of *Staphylococcus aureus*, potentially making it the dominant strain and triggering inflammatory diseases in the gut. Conversely, the abundance of *Lactobacillus johnsonii* YH1136 is inversely correlated with inflammatory responses, and these bacteria play a protective role in preventing inflammation induced by endogenous pathogenic bacteria in high-altitude environments (Wan et al., 2022). Several strains, including *Lactobacillus salivarius, Lactobacillus fermentum*, and *Lactobacillus rhamnosus*, have been shown to possess direct or indirect antimicrobial activity against *Staphylococcus aureus* (Kang et al., 2017; Liu G. et al., 2020). Furthermore, species such as *Bacillus proteolyticus* Z1 and Z2*, Bacillus amyloliquefaciens* J, and *Bacillus subtilis* K exhibit inhibitory effects against *Streptococcus enteritidis, Staphylococcus aureus*, and *Escherichia coli* (Zeng et al., 2021). Importantly, these strains have not tested positive for antibiotic resistance or hemolytic activity, indicating a favorable safety profile.

#### 3.2.4 Enhancement of xenobiotic detoxification

Gut microbiota can directly influence the metabolism and elimination of exogenous toxic compounds in the host, thereby modulating the toxic effects of these substances. For instance, it has been demonstrated that gut microbial species involved in the degradation of polycyclic aromatic hydrocarbons and toluene, such as *Streptomyces vietnamensis, Candidatus Formimonas warabiya*, and *Rhizobium leguminosarum*, are significantly enriched in rhesus monkeys residing at high altitudes compared to those at lower altitudes (Zhao et al., 2023b). Additionally, the expression of genes involved in the synthesis of L-cysteine, an amino acid known for its role in hepatic detoxification, was upregulated. L-cysteine is a pharmacological agent that protects hepatocytes from damage and facilitates the recovery of liver function (Yin et al., 2016). These findings suggest that, as part of their adaptation to high-altitude environments, the gut microbiota of rhesus monkeys plays a crucial role in detoxifying the host through metabolic processes. In conjunction with these mechanisms, the gut microbiota helps maintain immune homeostasis and mitigates the over-activation of immune responses during high-altitude exposure, thereby enhancing the host's adaptation to the plateau environment.

Overall, the gut microbiota orchestrates a multifaceted immunomodulatory program at high altitude that defends against environmental stress-induced oxidative damage and inflammatory injury. This coordinated defense is achieved through several complementary mechanisms: the intrinsic antioxidant capabilities of taxa such as *Lachnospiraceae*; the reinforcement of intestinal barrier integrity by *Akkermansia muciniphila* and microbiota-derived SCFAs; and the direct modulation of immune cell activity and inflammatory cytokine profiles by species including *bifidobacteria* and butyrate-producing *Firmicutes*. Future studies need to focus on identifying the molecular mechanisms associated with gut microbiota that mediate these effects, exploring their potential as immunomodulatory therapeutics in high-altitude environments.

### 3.3 Modulation of neuroendocrine signaling

As a potential endocrine organ, the gut microbiota produces various metabolites with signaling functions or hormonal properties, including SCFAs, neurotransmitters, neuroactive compounds, bile acids, and choline metabolites. Neurotransmitters such as gamma-aminobutyric acid (GABA; Strandwitz et al., 2019), dopamine (Omaliko et al., 2024), acetylcholine (Wu et al., 2023), norepinephrine (Cuesta et al., 2022), and 5-hydroxytryptophan (Liu et al., 2022) are secreted into the intestinal lumen by intestinal bacteria and can be transported through the bloodstream to distant effector organs (e.g., the brain). These signaling molecules mediate communication pathways through which the gut microbiota modulates the host's neuroendocrine system, forming what is commonly referred to as the gut-brain axis (Teichman et al., 2020) or the gut microbiota-metabolite-neuroendocrine axis (Wang J. et al., 2023). This system interconnects with host energy metabolism and immune function, establishing a complex multi-pathway network that enhances the host's ability to adapt to high-altitude environments by maintaining homeostasis. A classic example of this is the gut microbiota of high-altitude populations, which compensates for energy demands by increasing acetate production and activating the parasympathetic nervous system (Perry et al., 2016). This activation, in turn, promotes glucose-stimulated insulin secretion and enhances the release of growth hormone-releasing peptides, stimulating food intake and energy storage.

#### 3.3.1 Modulating neurotransmitters to alleviate depressive phenotypes

Previous studies have shown that high-altitude exposure may induce or exacerbate states of depression and anxiety in humans (Alcantara-Zapata et al., 2023). It is well-established that GABA is the primary inhibitory neurotransmitter in the brain, and its levels are closely linked to anxiety and depression. Transcriptomic analysis of human fecal samples from healthy individuals revealed that the GABA-producing pathways are actively expressed by bacterial genera such as *Bacteroides, Parabacteroides*, and *Escherichia*. It is speculated that *Bacteroides* may be the primary bacterial producers of GABA in the human gut. Additionally, studies combining 16S rRNA sequencing with functional magnetic resonance imaging of patients with major depressive disorder have shown that the relative abundance of fecal *Bacteroides* is negatively correlated with brain features associated with depression (Strandwitz et al., 2019). Similarly, experimental studies have demonstrated that GABA-producing *Lactobacillus* strains can reduce depression-like behaviors in mouse models of metabolic syndrome (Patterson et al., 2019).

#### 3.3.2 Reprogramming the renin-angiotensin system for blood pressure homeostasis

Additionally, the regulation of blood pressure is a key physiological response enabling the body to adapt to the reduced oxygen availability at high altitudes (Ivy and Scott, 2015). The neuroendocrine pathways, including the renin-angiotensin system, have been proposed as microbiota-mediated mechanisms for blood pressure regulation (Suzuki et al., 2019). The *Firmicutes phylum*, specifically the *Eubacterium* and *Roseburia*, are prominent producers of butyrate (Levy et al., 2016), while acetate and propionate are primarily produced by the *Bacteroidetes phylum* (Feng et al., 2018). These SCFAs, including acetate, propionate, and butyrate, activate various receptors such as G protein-coupled receptors (GPR) 41, GPR43, GPR109A, and olfactory receptors (OLFR) 78 and OLFR558 (Xu et al., 2022). These receptors regulate vascular contraction and dilation, playing a crucial role in controlling blood pressure. GPR41 is expressed in autonomic and sensory ganglia, as well as in the vascular endothelium of both mice and humans (Nohr et al., 2015), modulating vascular tone in response to hypotensive signals from SCFAs (Natarajan et al., 2016). In contrast, the activation of GPR109A by niacin induces Ca^2+^-dependent l-glutamate release, increasing oxidative stress in neurons of central blood pressure regulatory nuclei, leading to enhanced sympathetic excitability and elevated blood pressure (Rezq and Abdel-Rahman, 2016), which is essential for maintaining stable blood pressure levels. OLFR78 and OLFR558 are widely expressed in vascular smooth muscle cells of various organs (Pluznick et al., 2013), with OLFR558 being specifically enriched in renin-secreting cells (Brunskill et al., 2011). OLFR78 reduces the conversion of angiotensin I within the renin-angiotensin system by modulating renin secretion, thereby downregulating downstream pathways that elevate blood pressure, contributing to the regulation of pulmonary hypertension and adaptation to high-altitude environments. A cross-species study revealed that *Actinobacteria* and *Izhakiella* are commonly enriched in wild macaques, dogs, and humans at high altitudes (Zhao et al., 2023a). These taxa contribute to host adaptation to hypoxia through blood pressure modulation, yet the underlying causal mechanisms require elucidation.

In summary, synthesis of current evidence indicates that the gut microbiota acts as a key regulator of high-altitude adaptation, coordinating host physiology through integrated effects on energy metabolism, immune responses, and neuroendocrine signaling to maintain homeostasis (Table 1). However, the functional impact of this microbial remodeling is highly context-dependent, influenced by factors such as altitude gradient, exposure duration, host diet, and genetic background. Han and colleagues (Han et al., 2022) reported that the diversity and structure of the gut microbiota in rats exposed to hypobaric hypoxia undergo distinct dynamic changes over time, with experimental results suggesting day 5 as a potential tipping point for fundamental compositional shifts—implying that adaptation may occur through staged ecological recalibration. Besides, Jia et al. (2020) observed that individuals residing at high altitude retained microbial signatures even after returning to plain conditions for 3 months, suggesting that high-altitude exposure may induce long-term physiological reprogramming of the gut ecosystem. Elucidating the dynamics of this temporal microbial succession will be crucial to fully unravel the role of the gut microbiota in the adaptation process.

**Table 1:** Gut microbiota and metabolite profiles in high-altitude adaptation across species.

**Species**	**Study design and altitude exposure**	**Adaptive microbial shifts**	**Associated metabolites**	**Functional role in adaptation**	**Refs**
Tibetan chickens	Collected ileal and cecal contents from 12 Tibetan chickens living at high (3,572 m) and low altitudes (580 m), and then performed 16S rRNA sequencing.	*Bacteroides* ↑ *Peptoclostridium* ↑ *Ruminococcusou* ↑ *Bifidobacteriaceae* ↑ *Lactobacillus* ↑	–	Enhance host energy harvesting capacity and glycan biosynthetic pathways.	Du et al., 2022
Rhesus macaque	Collected 73 fecal samples from Rhesus macaques living at high (above 3,000 m) and low altitudes (below 500 m), and then performed 16S rRNA sequencing.	*Firmicutes* ↑ *Bacteroidetes* ↓ *Ruminococcace* ↑ *Prevotellaceae* ↓	Acetate ↑	Enhance host membrane transport and carbohydrate metabolic functions.	Wu et al., 2020
Nile tilapia	Collected 39 samples of intestinal contents from Nile tilapia at different altitudes (1,235–2,440 m), and then performed 16S rRNA sequencing.	F/B ratio ↑ *Fusobacteriota* ↑ *Actinobacteriota* ↓ *Chloroflexi* ↓ *Cyanobacteria* ↓	–	Promote carbohydrate degradation and efficient absorption of dietary energy.	Bereded et al., 2022
*Gymnocypris przewalskii*	Collected the gut contents of *Gymnocypris przewalskii* in Qinghai Lake (3,000 m) for 16S rRNA gene sequencing.	*Aeromonas* ↑ *Clostridium* ↑ *Cetobacterium* ↑ *Shewanella* ↑ *Vibrio* ↑ *Prochlorococcus* ↑	–	Enhance intestinal barrier integrity and inhibit inflammation.	Wang et al., 2021
Tibetan-antelopes, Tibetan-asses, Tibetan-sheep, and sheep	Collected 22 fecal samples from 9 Tibetan-antelopes, 3 Tibetan-asses, and 4 Tibetan-sheep living at 4,300 m, 3 Tibetan-sheep living at 3,000 m, 3 sheep living at 1,800 m, and then performed 16S rRNA sequencing.	*Ruminococcus* ↑ *Oscillospira* ↑ *Clostridium* ↑	–	Upregulate carbohydrate metabolism and optimize dietary energy harvest.	Ma et al., 2019
C57BL/6 mice	Randomly divided the 36 mice into three groups: control group (94.5 kPa, physiological saline), high altitude group (3,500–4,000 m for 14 days, 60–65 kPa, physiological saline), high-altitude probiotic group (3,500–4,000 m for 14 days, 60–65 kPa, probiotic). Collected samples and subjected them to 16S rRNA sequencing.	*Lactobacillus* ↑ *Staphylococcus spp*. ↓	–	Suppress the inflammatory response induced by the endogenous pathogen.	Wan et al., 2022
Tibetan yaks	Collected fecal samples from the yaks of Tibet (4,000 m), then isolated and identified the strains with excellent probiotic potential from the samples.	*Bacillus proteolyticus* Z1 and Z2 ↑ *Bacillus amyloliquefaciens* J ↑ *Bacillus subtilis* K ↑	–	Inhibit the activity of *Streptococcus enteritidis* NTNC13349, *Streptococcus aureus* BNCC186335, and *Escherichia coli* C83902.	Zeng et al., 2021
Rhesus macaques	Compared the composition and functional differences of gut microbiota in wild rhesus macaques from high (above 3,500 m) and low altitudes (below 1,000 m) through metagenomic and metabolomic analyses.	*Streptomyces vietnamensis* ↑ *Tsukamurella paulometabola* ↑ *Nocardia brasiliensis* ↑ *Formimonas warabiya* ↑ *Rhizobium leguminosarum* ↑	Acetate ↑	Metabolize and remove exogenous toxic compounds (cyclic aromatic hydrocarbons, toluene); up-regulate the gene expression of L-cysteine and promote liver detoxification metabolism.	Zhao et al., 2023b
Humans, pigs, Rex rabbits, and Rhesus macaques	Collected fecal samples from 441 humans (below 50 m ~ above 3,750 m), 102 pigs (below 100 m ~ above 3,750 m), 34 Rex rabbits (478 m), and 12 Rhesus macaques (below 100 m ~ above 3,000 m); and then performed 16s RNA, metagenomic sequencing analysis, and metabolomic studies.	*Acinetobacter* ↑ *Pseudomonas* ↑ *Sphingobacterium* ↑	Propanoic acid ↑ Octadecanoic acid ↑	Promote host energy metabolism, amino acid metabolism, and carbohydrate metabolism.	Zeng et al., 2020
Healthy male adults traveling from Chongqing (243 m) to Lhasa (3,658 m) and back	Performed metabolomics analysis of the 46 fecal samples collected at seven time points over 108 days in the longitudinal cohort	*Collinsella* ↑ *Blautia A* ↑ *Enterocloster* ↑	Uric acid ↓ Butyrate ↑	Regulate intestinal uric acid metabolism and promote high-altitude hypoxia adaptation.	Su et al., 2024a
Tibetan and Han populations at the Qinghai-Tibetan Plateau and low-altitudes	Obtained 668 original 16S rRNA sequencing data of Tibetan and Han populations at the Qinghai-Tibetan Plateau and low-altitudes from public databases; collected 44 fecal samples from Han volunteers at high-altitude (*n* = 40) and low-altitude (*n* = 4). Then, a meta-analysis on the acquired public data, and 16S rRNA sequencing on the collected fecal samples.	*Dorea* ↑ *Roseburia* ↑ *Prevotella 9* ↑ *Faecalibacterium* ↑	Butyrate ↑	Reduce LDHA activity and down-regulate HIF-1α expression to alleviate hypoxic stress and reduce intestinal injury caused by high altitude exposure.	Zhao et al., 2024
Human, dog, wild macaque, and coyote	Collected 152 fecal samples of human (*n* = 40), dog (*n* = 40), wild macaque (*n* = 54), and coyote (*n* = 18) from high-altitude (>3,000 m) and low-altitude (< 1,000 m) environments, then performed 16s rRNA sequencing.	*Actinomycetes* ↑ *Alloprevotella* ↑	Acetate ↑ Succinate ↑	Regulate blood pressure and promote host adaptation to the hypobaric hypoxia environment.	Zhao et al., 2023a
Hypertensive patients and healthy individuals	Collected the fecal samples among hypertensive patients and healthy individuals from 3 distinct altitudes, including Tibetans at high altitude (3,600-4,500 m, *n* = 38 and 34), Hans at middle altitude (2,260 m, *n* = 49 and 35), and Hans at low altitude (13 m, *n* = 34 and 35), then performed 16S rRNA sequencing.	*Akkermansia* ↑	–	Promote hypertension regulation at high altitude.	Zhu et al., 2020

This table summarizes studies reporting specific microbial and metabolite alterations associated with high-altitude adaptation in various animal models and human cohorts. For each study, the subject/species, experimental design and altitude exposure, key microbial changes (with directional shifts indicated by ↑/↓), associated metabolites, functional role in adaptation, and corresponding references are provided. SCFAs, short-chain fatty acids; F/B ratio, *Firmicutes/Bacteroidetes* ratio; C57BL/6 mice, C57 Black 6 mice; Refs, References.

## 4 Gut microbiota homeostatic disruption in high-altitude disorders

**Figure 2:**
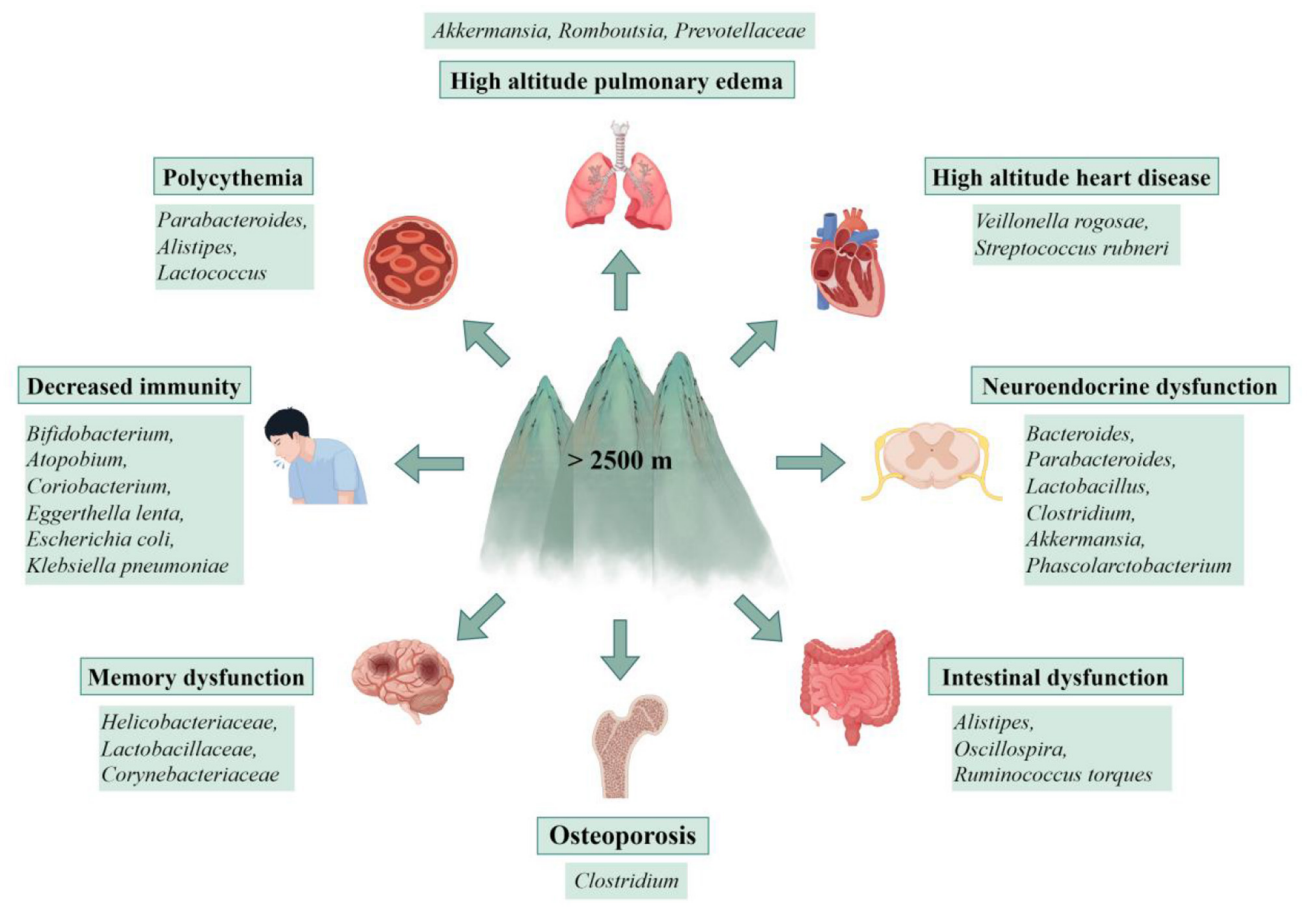
Gut microbiota dysbiosis contributes to multi-system disorders at high altitude. Disruption of gut microbial homeostasis under high-altitude stress is associated with an increased risk of disorders across multiple organ systems, including high altitude pulmonary edema, high altitude heart disease, polycythemia, decreased immunity, neuroendocrine and memory dysfunction, intestinal disorders, and osteoporosis. This figure presents the connections between a dysbiotic gut community and the pathogenesis of these conditions, highlighting the gut microbiota's role as a key interface between environmental stress and host health (by Figdraw ID: WPORU741bb.).

Growing evidence indicates that acute exposure to high altitudes impairs the intestinal barrier through mechanisms involving hypoxia and oxidative stress. Prolonged hypoxic exposure may further compromise the intestinal barrier by disrupting immune function, altering microbial community composition, and damaging the mucosal layer, leading to endotoxemia and low-grade intestinal or systemic inflammation (McKenna et al., 2022). These changes can manifest with symptoms affecting the digestive, respiratory, cardiovascular, immune, and nervous systems (Figure 2). Dysbiosis of the gut microbiota induced by acute high-altitude exposure is closely associated with the onset of acute high-altitude illnesses, such as AMS, HACE, and HAPE. In cases where high-altitude adaptability and compensatory mechanisms are overwhelmed, CMS may develop, including conditions such as chronic high-altitude polycythemia, high-altitude heart disease, high-altitude hypertension, and mixed high-altitude disease (Penaloza and Arias-Stella, 2007).

Song et al. (2024) established a mouse model of acute high-altitude hypoxia and found that abrupt exposure to the harsh environment caused gut microbiota dysbiosis and widespread inflammatory cell infiltration, leading to pulmonary edema, cerebral edema, alveolar hemorrhage, and damage to the intestinal barrier. The F/B ratio, as well as the relative abundances of *Romboutsia* and *Prevotella*, were identified as key factors influencing these pathological changes. Although much of the current research focuses on the mechanisms of gut microbiota adaptation to high-altitude environments and the pathogenesis of chronic high-altitude diseases across various species, the strong association between the onset and progression of acute high-altitude illness and gut microbiota dysbiosis should not be overlooked. With recent advances in the study of the “gut-brain” and “gut-lung” axes (Budden et al., 2017; Zhuang et al., 2024), the pathological mechanisms of acute high-altitude illnesses mediated by gut microbiota are likely to be reinterpreted in light of these emerging concepts.

Under the challenge of hypobaric hypoxia induced by long-term high-altitude exposure, abnormalities in the gut microbiota and microbial metabolites can lead to high-altitude heart disease and high-altitude polycythemia in rats, primarily driven by bacterial families such as *Prevotellaceae, Porphyromonadaceae*, and *Streptococcaceae* (Pan et al., 2022). Recent research indicates (Zhou et al., 2025)that reduced abundances of *Veillonella rogosae* and *Streptococcus rubneri* remodel metabolic processes in high-altitude migrants, thereby increasing susceptibility to high-altitude cardiac health abnormalities, which are associated with hypobaric hypoxia-induced myocardial injury and cardiac hypertrophy. Furthermore, Wang et al. (2022) found significantly increased relative abundances of *Alistipes, Acetatifactor*, and *Akkermansia muciniphila* in the feces of mice exposed to high-altitude environments. Notably, the increased abundance of *Alistipes* has been positively correlated with diseases such as intestinal inflammation, irritable bowel syndrome (Zhang et al., 2023b), depression (Parker et al., 2020), and chronic fatigue syndrome (Nagy-Szakal et al., 2017). On the other hand, a higher abundance of *Akkermansia muciniphila* has been identified as a potential risk factor for increased susceptibility to colitis in mice (Khan et al., 2020). Kleessen et al. (2005) observed that the fecal microbiota of mountaineers exposed to altitudes above 5,000 m showed a decline in *Bifidobacterium, Atopobium, Coriobacterium*, and *Eggerthella lenta*, along with increased levels of opportunistic pathogens like *Escherichia coli* and *Klebsiella pneumoniae*. This shift in microbial composition was accompanied by reduced serum levels of IgM and/or IgA, as well as significantly elevated C-reactive protein levels, suggesting a link between gut microbiota dysbiosis and impaired immune function in individuals acutely exposed to high altitudes. Machado et al. (2017) further noted that exercise under hypoxic conditions at high altitudes can compromise the integrity of the intestinal mucosal barrier, leading to a significant increase in plasma endotoxin levels and the onset of inflammatory diseases, including irritable bowel syndrome, endotoxemia, and pulmonary hypertension. A cohort study also reported that high-altitude exposure leads to intestinal mucosal barrier damage and the release of pro-inflammatory cytokines, such as TNF-α and IL-1β (Zhang Y. W. et al., 2024), which may increase osteoclast number and activity, thereby reducing bone density and increasing the risk of osteoporosis. *Clostridium* has been implicated in mediating this process (Zuo et al., 2022).

In recent years, with advances in understanding the microbiota-gut-brain axis, it has been found that homeostatic imbalances in gut microbiota and their metabolites (e.g., SCFAs and bile acids) can induce neurological dysfunction (Teichman et al., 2020). High-altitude environments significantly affect the richness and diversity of gut microbiota, leading to microbial community restructuring. The dysregulation of *Helicobacter pylori* may further exacerbate working memory impairment induced by high-altitude exposure (Zhao Z. et al., 2022). Zhang et al. (2023a) demonstrated that high-altitude exposure increases gut permeability, decreases ileal immune function, and reduces both the richness and diversity of the ileal microbiota in mice. Specifically, the dysregulation of *Lactobacillaceae* ASV78 and *Lachnospiraceae* ASV25, ASV47, and ASV78 was associated with exacerbated spatial memory impairment caused by high-altitude exposure. Kadry and Megeed (2018) found that *Lactobacillaceae* effectively mitigated the reduction of hippocampal brain-derived neurotrophic factor caused by cadmium chloride in a toxicity mouse model, a finding that was positively correlated with memory preservation. Additionally, Corpuz et al. (2018) confirmed that *Lactobacillus paracasei K71* could prevent accelerated cognitive decline in aging mice by upregulating hippocampal brain-derived neurotrophic factor protein expression and serotonin levels. In contrast, Lachnospiraceae has been shown to have the opposite effect, potentially exacerbating inflammatory responses and impairing spatial memory, which correlates with reduced cognitive function (Zhi et al., 2017). Acute high-altitude exposure also triggers hyperactivation of the hypothalamic-pituitary-adrenal and hypothalamic-pituitary-thyroid axes (Wang J. et al., 2023), leading to neuroendocrine dysfunction. This is associated with the enrichment of bacterial genera such as *Bacteroides, Parabacteroides, Lactobacillus, Clostridium, Akkermansia*, and *Collinsella*.

Cumulative evidence links gut microbiota dysbiosis to various acute and chronic high-altitude pathological conditions (Table 2). Nevertheless, a more complex challenge facing the field lies in advancing from these correlative observations toward a mechanistic understanding. Current research often stops at identifying disease-associated microbial families or genera, without elucidating the functions of specific bacterial species/strains or the underlying pathways involved. The interventional strategies discussed in the following chapter represent concerted efforts to therapeutically target this dysbiotic state for improved disease outcomes.

**Table 2:** Specific dysbiotic gut microbiota signatures in high-altitude maladaptation.

**Study subjects**	**Study protocol**	**Key microbial alterations**	**Associated metabolites**	**High-altitude maladaptation**	**Refs**
48 male specific pathogen-free C57BL6/J mice	Mice were raised in a hypoxic environment simulating an altitude of 7,000 m (oxygen concentration of 8.5%); 7 days. Then, lung and brain organ water content measurement, histopathological analysis, ileal tissue cytokine measurement, and antioxidant capacity assessment, as well as 16S rRNA sequencing analysis of stool samples.	F/B ratio ↑ *Akkermansia* ↑ *Romboutsia* ↑ *Prevotellaceae* UCG-001 ↑	–	High altitude pulmonary edema, high altitude cerebral edema.	Song et al., 2024
20 male Wistar rats	Rats were randomly divided into a normobaric normoxia group and a hypobaric hypoxia group (atmospheric pressure of 53.8 kPa with an oxygen partial pressure of 11.3 kPa, simulating exposure to an altitude of 5,000 m); 28 days. Then, monitored erythrocyte indices, myocardial histopathology, gut microbiota composition by 16s RNA sequencing, and levels of SCFAs and bile acids by metabolomics analysis.	*Parabacteroides* ↑ *Alistipes* ↑ *Lactococcus* ↑	Propionate ↓ Bile acids ↓	High altitude heart disease, high altitude polycythemia.	Pan et al., 2022
230 adult males	Enrolled two groups: plain group comprising 67 adult males from the Chengdu plain in Sichuan, and high-altitude group comprising 163 adult males from Tibet (3,500–4,500 m). Monitored the heart health of all subjects; collected fecal samples for metagenomic sequencing and metabolomics analysis.	*Veillonella rogosae* ↓ *Streptococcus rubneri* ↓	L-aspartic acid ↓ Betaine ↓ɑ-ketoglutaric acid ↓	High altitude heart disease.	Zhou et al., 2025
122 Han Chinese males and 25 Tibetan males	122 Han Chinese males from low-altitude regions (100–1,000 m), divided into a high-altitude group (*n* = 92) relocated to Lhasa (3,650 m) and a low-altitude control group (*n* = 30) moved to Tianjin (10–100 m); 25 Tibetan males from Tibetan Plateau regions (3,000–4,500 m) were relocated to Lhasa. Evaluated the participants' clinical symptoms. Collected fecal samples at 3, 6, and 12 months for 16S rRNA gene sequencing.	*Alistipes* ↑ *Oscillospira* ↑ *Ruminococcus torques* ↑	–	Intestinal inflammation, irritable bowel syndrome, endotoxemia, depression, and chronic fatigue syndrome.	Zhang et al., 2023b
5 male and 2 female mountaineers	Investigated fecal samples and serum of 7 mountaineers who completed a 47-day expedition to the Nepal Himalayas (5,000 m). Detected the gut microbiota in stool samples by fluorescence *in situ* hybridization and epifluorescence microscopy, and assessed the level of immune response by ELISA.	*Bifidobacterium* ↓ *Atopobium* ↓ *Coriobacterium* ↓ *Eggerthella lenta* ↓ *Escherichia coli* ↑ *Klebsiella pneumoniae* ↑	–	Immunocompromised.	Kleessen et al., 2005
73974 adults from five provinces of Southwest China	Collected tool samples (*n* = 1,384) for 16S rRNA gene sequencing, and then assessed the association between altitude and bone mineral density using multivariate linear regression models and mediation analyses, with a focus on the potential mediating role of gut microbiota.	*Clostridium* ↑	Acetic ↓ Propionic ↓ Butyric ↓	Osteoporosis.	Zuo et al., 2022
36 C57BL/6 mice	Mice were randomly divided into three groups: control group (94.5 kPa); high-altitude exposed group (3,500–4,000 m, 60–65 kPa); high-altitude exposed with antibiotic treatment group (3,500–4,000 m, 60–65 kPa, 0.2 g/L of ciprofloxacin and 1 g/L of metronidazole); 14 days. Determined the antioxidant capacity in the prefrontal cortex and tested the working memory function of all mice. Collected tool samples for 16S rRNA gene sequencing.	*Helicobacteriaceae* ↑	–	Working memory disorders.	Zhao Z. et al., 2022
36 C57BL/6 mice	Mice were randomly divided into three groups: control group (94.5 kPa); high-altitude exposed group (4,000 m, 60–65 kPa); high-altitude exposed with antibiotic treatment group (4,000 m, 60–65 kPa, 0.2 g/L of ciprofloxacin and 2 g/L of metronidazole); 14 days. Tested the memory function and detected the expression of memory-related proteins. Collected tool samples for 16S rRNA gene sequencing.	*Lactobacillaceae* ASV11 ↓ *Corynebacteriaceae* ASV78 and ASV25 and ASV47 ↓	–	Spatial memory disorders.	Zhang et al., 2023a
12 male Sprague-Dawley rats	The hypoxia group (*n* = 6) was placed in a hypobaric oxygen chamber that mimics an altitude of 5,500 m (379 mmHg), while the control group (*n* = 6) was kept in a normoxic environment (Beijing, China, 52 m, 760 mmHg); 3 days. Then performed the ELISA and metabolomic analyses of serum and 16S rRNA and metabolomic analyses of fecal samples.	*Bacteroides* ↑ *Parabacteroides* ↑ *Lactobacillus* ↑ *Clostridium* ↑ *Akkermansia* ↑ *Phascolarctobacterium* ↑	Prenol lipids ↑ Glycerophospholipids ↓ Steroids ↑	Neuroendocrine dysfunction, excessive activation of the hypothalamic-pituitary-adrenal axis and hypothalamic-pituitary-thyroid axis.	Wang J. et al., 2023

This table compiles evidence linking gut microbiota dysbiosis to various high-altitude pathologies. It details the model/cohort, experimental or observational protocol, key microbial alterations (with increased/decreased abundance indicated by ↑/↓), associated maladaptation syndromes, and references. F/B ratio, *Firmicutes/Bacteroidetes* ratio; C57BL/6J mice, C57 Black 6 Jackson Laboratory mice; Refs, References.

## 5 Exploration of strategies for modulating gut microbiota to prevent and treat high-altitude disorders

The “Consensus Guidelines for the Prevention and Treatment of Acute Mountain Sickness” indicate that gradual ascent is the most effective method for preventing all forms of altitude sickness (Luks et al., 2024). In terms of pharmacological interventions, acetazolamide, analgesics, and dexamethasone are commonly administered to prevent or treat mild AMS. Acetazolamide primarily functions by inhibiting carbonic anhydrase in the kidneys, red blood cells, and other tissues, thereby preventing metabolic acidosis. Its diuretic effect also mitigates water and sodium retention, alleviating symptoms associated with HAPE and HACE. A clinical trial has shown that acetazolamide effectively increases the mean pulmonary artery flow acceleration time while reducing the pulmonary vascular resistance index in patients with CMS (Leon-Velarde et al., 2010). Nonsteroidal anti-inflammatory drugs, such as ibuprofen, are frequently utilized by travelers to high altitudes to alleviate symptoms of AMS and high-altitude headaches. Dexamethasone, a glucocorticoid, is typically employed to manage severe manifestations of AMS, HACE, and HAPE. The most common side effects of these three medications include gastrointestinal discomfort and an elevated risk of gastrointestinal bleeding; however, their specific impact on gut barrier integrity during high-altitude exposure remains uncertain.

**Figure 3:**
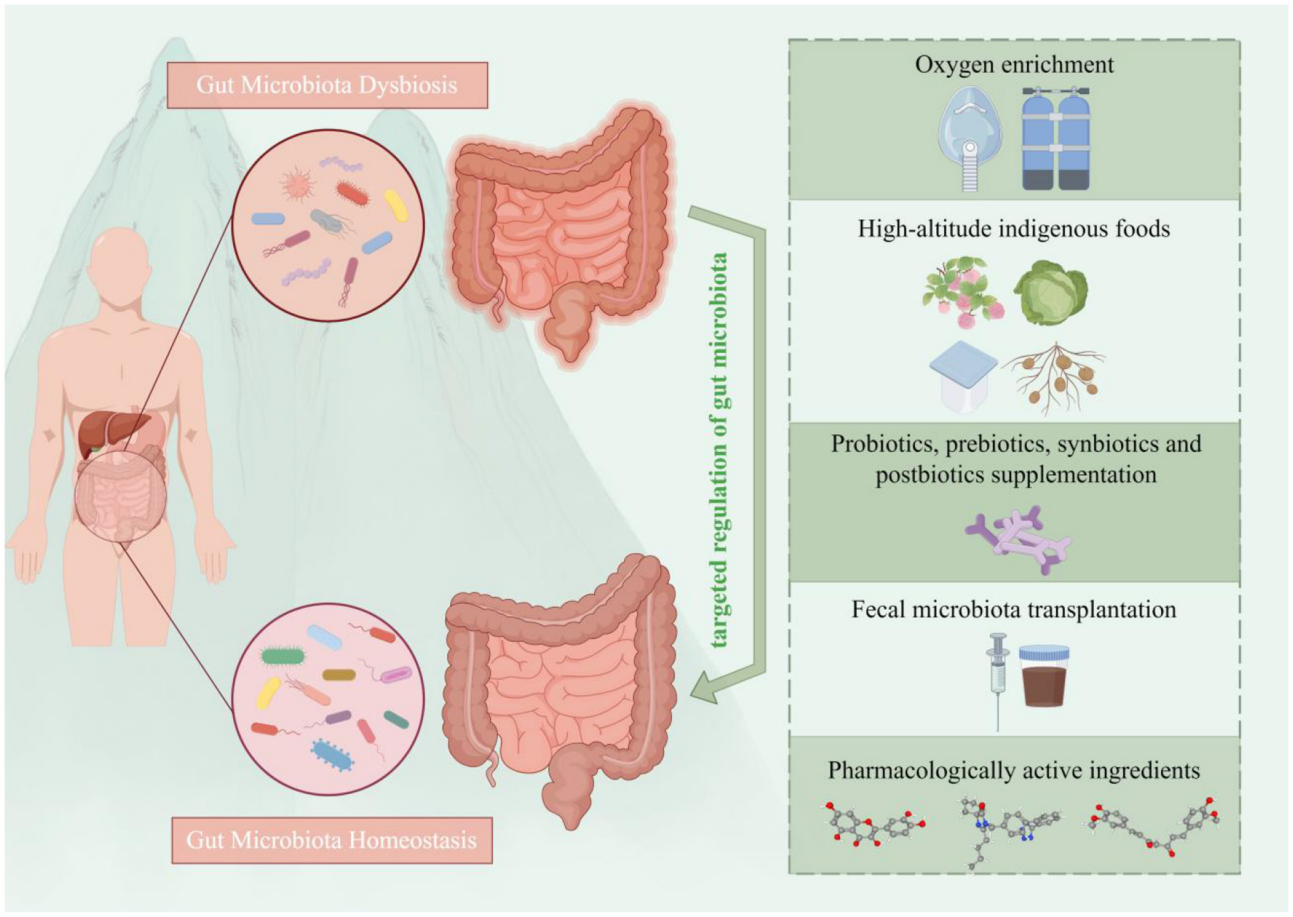
Overview of gut microbiota-targeted strategies for the prevention and treatment of high-altitude illnesses. Five main categories of interventions are depicted: (i) environmental modulation: via oxygen enrichment; (ii) nutritional strategies: the consumption of indigenous fermented foods (e.g., berries, *Brassica rapa* L., Tibetan fermented milk and resistant potato starch); (iii) microbial supplementation: using probiotics, prebiotics, synbiotics, or postbiotics; (iv) microbiota restoration: via fecal microbiota transplantation; and (v) pharmacological intervention: employing bioactive compounds (e.g., quercetin, citrus tangerine pith extract). These strategies aim to prevent and manage high-altitude illnesses by rectifying dysbiosis and restoring microbial homeostasis (by Figdraw ID: WPSTAca260.).

Current research indicates that dysbiosis of the gut microbiota plays a significant role in the development of high-altitude disease states. Consequently, exploring and developing prevention or treatment strategies for high-altitude illnesses that specifically target the gut microbiota and their associated metabolites holds considerable promise. These approaches may offer a distinct advantage over traditional pharmacological treatments by circumventing gastrointestinal discomfort as a side effect. The subsequent sections will review effective interventions (Figure 3).

### 5.1 Oxygen enrichment

The low-pressure hypoxic environment at high altitudes is a direct factor that induces varying degrees of altitude-related reactions and high-altitude diseases. It is well-established that oxygen supplementation can alleviate hypoxia, promote the rebalancing of physiological functions, and facilitate acclimatization to high-altitude conditions. Previous studies have demonstrated that oxygen-enriched technology can provide a sustained oxygen supply, thereby improving right ventricular hypertrophy and myocardial cell hypertrophy caused by chronic high-altitude hypoxia. This is achieved by inhibiting the overexpression of cytokines such as angiotensin-converting enzyme/angiotensin-converting enzyme 2, angiotensin II, type I collagen α, type III collagen α1, hydroxyproline, and endothelin-1. In addition, oxygen-enriched interventions have been shown to suppress the increase in erythropoiesis induced by high-altitude hypoxia (Shao et al., 2021).

Recent evidence indicates that oxygen-enriched interventions significantly attenuate gut injury caused by acute high-altitude hypoxia. This is characterized by increased crypt depth, intact epithelial cell morphology, a higher relative density of goblet cells, and enhanced uniformity and richness of *Prevotellaceae* NK3B31 and *Ruminococcus* in the gut microbiota (Ma et al., 2023). Under this altered gut microbiota structure, butyrate concentrations were elevated, contributing to improved energy and amino acid metabolism. This provides a more sufficient energy source for colon cells and plays a beneficial role in reducing inflammatory responses, mitigating brain dysfunction (Mishra et al., 2024), and improving heart failure (Modrego et al., 2023). Furthermore, oxygen-enriched interventions have demonstrated clear benefits in enhancing neuropsychological functions in high-altitude populations, including improved hand-eye coordination, memory, cognitive function, and overall psychological wellbeing (Moraga et al., 2018). Given the previously discussed positive regulatory effects of the gut microbiota on the host's neuroendocrine system, we hypothesize that there may be unexplored connections between these two factors.

Collectively, oxygen enrichment at high altitude appears to mitigate hypoxic damage by simultaneously safeguarding gut integrity and promoting a healthier gut microbial ecosystem. This coordinated response enhances overall physiological adaptation and reduces susceptibility to altitude illnesses.

### 5.2 High-altitude indigenous foods

The composition and functional activity of the gut microbiota are modulated by dietary macronutrient intake (David et al., 2014). For instance, high-fiber diets correlate with increased abundances of probiotic genera such as *Bacteroides* and *Bifidobacterium*, which help modulate host immune function and suppress local and systemic inflammatory responses, thereby reducing the incidence of respiratory and cardiovascular diseases in high-altitude environments (Jang et al., 2021; Zhao et al., 2022). Ketogenic diets enhance neurovascular protective functions by elevating *Akkermansia muciniphila* and *Lactobacillus* populations while reducing *Desulfovibrio* abundance (Ma et al., 2018). Conversely, high-fat diets are strongly associated with the overgrowth of endotoxin-producing pathogens like *Enterobacter* (Wu et al., 2011). Our analysis focuses on several healthy foods native to high-altitude regions, which exert critical supportive effects on host altitude adaptation through microbiota-mediated mechanisms.

Burlando et al. (2023) have highlighted that certain berries, such as high-latitude cloudberries (Rubus chamaemorus) and Arctic brambles (Rubus arcticus), as well as high-altitude yellow Himalayan raspberries (Rubus ellipticus), have significant potential as nutritional health supplements in high-altitude regions. These berries are rich in ellagitannins, potent antioxidants, and bioactive compounds. Natural metabolites, such as urolithin, produced through the fermentation of these berries by the gut microbiota, exhibit anti-inflammatory, antioxidative, antiviral, antibacterial, anti-aging, neuroprotective, and cardiovascular protective properties (Tow et al., 2022). These benefits are crucial in preventing high-altitude-related illnesses (e.g., upper respiratory tract infections) and alleviating symptoms of high-altitude sickness, including gastrointestinal disturbances (nausea, vomiting), neuropsychological symptoms (fatigue, drowsiness), and circulatory issues (headache, dizziness, palpitations, shortness of breath, cyanosis of the lips and fingers, and elevated heart rate).

*Brassica rapa* L., a natural food and economic crop thriving in high-altitude environments, contains polysaccharides as its primary bioactive components, which exhibit anti-hypoxic, anti-fatigue, glucolipid metabolism-regulating, and immunomodulatory properties (Hamed et al., 2024). Studies demonstrate that intervention with *Brassica rapa* L. crude polysaccharides enriches beneficial gut bacteria (e.g., *Leuconostoc lactis* and *Akkermansia muciniphila*) and elevates butyrate levels, enhancing intestinal barrier integrity, modulating amino acid biosynthesis pathways and antioxidant enzyme activities, thereby alleviating high-altitude exposure-induced oxidative stress damage and inflammatory responses (Liu et al., 2024).

Furthermore, the study suggests that the long-term consumption of Tibetan fermented milk enhances gut microbial diversity, increases the relative abundance of *Bacteroides* and *Faecalibacterium* (Liu J. et al., 2020), reduces amyloid-β deposition in the cerebral cortex and hippocampus, and improves cognitive impairment in animal models. This indicates potential therapeutic effects in preventing and treating altitude-induced cognitive decline in high-altitude environments.

Supplementation with fermented fiber derived from potato tubers significantly increases the relative abundance of *Bifidobacterium, Ruminococcus*, and *Clostridium* in fecal samples, which in turn elevates the average concentrations of butyrate (Baxter et al., 2019). This butyrate production contributes to alleviating inflammatory bowel disease, promoting the repair of gut mucosal barriers, and enhancing nutrient absorption and energy utilization in hosts living in high-altitude environments.

### 5.3 Probiotics, prebiotics, synbiotics, and postbiotics supplementation

Probiotics are a class of active microorganisms that confer health benefits to the host (King et al., 2021). These microorganisms can be introduced into the gut via external supplementation, where they interact synergistically with the host's resident microbiota. A growing body of evidence supports the role of probiotics as beneficial nutritional supplements for athletes, with studies indicating their ability to alleviate fatigue and mitigate environmental stress, particularly in individuals residing at high altitudes (McKenna et al., 2022). The mechanisms through which probiotics exert their effects are multifaceted, encompassing optimization of gut microbiota composition, enhancement of beneficial bacterial populations, protection of the intestinal barrier, inhibition of pathogen invasion to prevent endotoxemia, reduction of oxidative stress, modulation of immune responses, suppression of pro-inflammatory cytokines, promotion of anti-inflammatory cytokines, and augmentation of the production of beneficial metabolites (DeGruttola et al., 2016).

Prominent examples of probiotic supplements include the genera *Lactobacillus* and *Bifidobacterium*. West et al. (2011) in a randomized controlled trial, demonstrated that supplementation with fermented *Lactobacillus* increased the fecal probiotic counts by 7.7-fold in male athletes and 2.2-fold in female athletes. Song et al. (2024) reported that *Lactobacillus bulgaricus* 4L3 significantly downregulated hypoxia-induced increases in IL-6 and TNF-α, while elevating the levels of superoxide dismutase. This contributed to the reduction of inflammatory responses and oxidative stress damage, effectively mitigating hypoxia-induced cerebral and pulmonary edema. Additionally, it increased the expression of tight junction proteins in the ileum, reduced intestinal permeability, and alleviated mechanical barrier damage caused by acute hypoxia. Boris et al. (2001) highlighted the ability of *Lactobacillus delbrueckii subsp. lactis* UO004 to inhibit pathogens such as *Escherichia coli, Pseudomonas aeruginosa, Staphylococcus aureus*, and *Salmonella typhimurium*. Supplementation with *Lactobacillus plantarum* HEAL9 and/or *Lactobacillus paracasei* N1115 has been shown to effectively prevent acute upper respiratory tract infections in various populations (Zhao Y. L. et al., 2022). In high-altitude regions, *Lactobacillus plantarum* S27 has been proposed as a potential alternative to antibiotics in bird feed (Benbara et al., 2020). Furthermore, animal and *in vitro* studies have demonstrated that *Lactobacillus acidophilus* DDS-1 can enhance mucosal barrier function by upregulating mucin gene expression and promoting mucin secretion from intestinal epithelial cells, while simultaneously secreting antimicrobial peptides (Mack et al., 2003). These findings suggest that *Lactobacillus* supplementation may reduce the risk of pathogenic infections. Long-term consumption of *Lactobacillus fermentum* CECT5716 has also been found to ameliorate vascular oxidative stress and pro-inflammatory states, resulting in lowered systolic blood pressure in spontaneously hypertensive rat models, thereby providing cardiovascular protection (Gomez-Guzman et al., 2015). Similarly, studies have shown that supplementation with *Lactobacillus* probiotics during antibiotic treatment can restore bone marrow cell populations and increase SCFAs levels—particularly acetate, butyrate, and propionate (Tang et al., 2019)—as well as reduce circulating leptin levels, alleviate cardiac hypertrophy and remodeling post-myocardial infarction, promote recovery of post-ischemic mechanical function (Lam et al., 2012), and yield cardioprotective effects. Of note, recent research has suggested that the genera *Eubacterium, Roseburia*, and *Faecalibacterium* may also qualify as probiotics (Gibson et al., 2017); however, direct clinical evidence regarding their role in the prevention and treatment of high-altitude sickness is lacking.

Prebiotics are substrates that promote the growth and activity of probiotics. While prebiotics themselves are not absorbed by the human body and do not exert direct physiological effects, they selectively stimulate the growth and metabolic activity of beneficial microbes in the digestive tract. Prebiotics commonly consist of indigestible oligosaccharides, which are fermented by gut microbiota, resulting in the increased production of beneficial SCFAs. For instance, a study investigating the effects of SCFAs on hypertension-induced cardiac damage and atherosclerosis demonstrated that propionate administration significantly reduced cardiac hypertrophy, fibrosis, vascular dysfunction, and hypertension in mice (Bartolomaeus et al., 2019). Inulin is one of the most widely studied and effective prebiotics. *In vivo* studies have shown that ingestion of inulin-type fructooligosaccharides can reduce the F/B ratio, increase the abundance of *Bifidobacterium* and *Lactobacillus*, and inhibit the invasion of pathogens into intestinal cells. These effects contribute to the maintenance of intestinal barrier integrity, reduce pathogen-induced infections, and optimize host metabolic capacity (Ding et al., 2021). Various foods are recognized as sources of prebiotic carbohydrates, including chickpeas, purslane, broad beans, lentils, bean seeds, chicory root, onions, artichokes, garlic, tomatoes, and asparagus (Vaz Rezende et al., 2021).

Synbiotics are combinations of probiotics and prebiotics that work synergistically to enhance health benefits. Research by Khanna et al. (2021) showed that synbiotics can significantly reduce pro-inflammatory cytokines and chemokines, alleviate intestinal inflammation and gut dysbiosis caused by high-altitude hypoxia, and improve gut barrier function. These findings suggest that synbiotics hold significant promise as a strategy for preventing and treating digestive symptoms associated with high-altitude sickness. Further experiments from Ren et al. (2024) confirmed that the combination of stachyose and *Lactobacillus rhamnosus GG* was more effective than either stachyose or *Lactobacillus rhamnosus GG* alone in modulating gut barrier dysfunction induced by acute hypoxia. This combination significantly lowered the F/B ratio, increased butyrate levels, elevated anti-inflammatory cytokines (transforming growth factor-β) and antioxidant enzymes (superoxide dismutase, catalase, glutathione peroxidase), and decreased the expression of pro-inflammatory cytokines and hypoxia-inducible factors (Interferon-γ, IL-1β, IL-6, TNF-α, and HIF-1α), thereby protecting gut barrier function. It is speculated that this synbiotic combination may be an ideal intervention for preventing and treating intestinal dysfunction in high-altitude environments. Hu et al. (2022) reported that administration of probiotics (28 strains of *Bifidobacterium* and 8 strains of *Lactobacillus*), prebiotics (polydextrose, galactooligosaccharides, and inulin), and synbiotics (a mixture of probiotics and prebiotics) significantly reduced cardiac hypertrophy induced by prolonged hypobaric hypoxia exposure. Additionally, these interventions ameliorated hypobaric hypoxia-induced alterations in the gut microbiome and metabolome.

Postbiotics are bioactive compounds derived from the metabolic processes of probiotics, including SCFAs, iso-butyrate, lactate, and palmitoylethanolamide. Butyrate is a well-studied postbiotic, known for its beneficial effects on various metabolic disorders. Administration of butyrate has been shown to improve non-alcoholic fatty liver disease and increase the abundance of butyrate-producing bacteria such as *Eubacterium hallii, Christensenellaceae*, and *Lactobacillus* (Zhou et al., 2017). While most clinical research on postbiotics has focused on metabolic diseases, the potential role of postbiotics in preventing and treating high-altitude sickness warrants further investigation.

### 5.4 Fecal microbiota transplantation

Fecal microbiota transplantation (FMT) involves the transfer of functional microbiota from the feces of healthy donors into the gastrointestinal tract of individuals with dysbiosis, aiming to reconstruct gut microbiota and treat both gastrointestinal and extra-intestinal diseases. As a novel and effective approach distinct from dietary interventions and probiotic supplementation, FMT directly alters the gut microbiota composition and has been explored for the treatment of various microbiota-related disorders, including *Clostridium difficile* infection (Kelly et al., 2016).

Recent evidence suggests that intragastric administration of *Blautia wexlerae* to a mouse model exposed to high-altitude hypoxia significantly increased arterial oxygen partial pressure, improved pulmonary arterial pressure, right ventricular systolic pressure, and lung injury scores. Additionally, it was observed that the expression of pro-inflammatory cytokines IL-1α and IL-1β in the ileum was reduced, along with decreases in collagen fiber deposition in the lungs, pulmonary edema, and ileal tissue damage (Su et al., 2024b). These findings indicate that *Blautia wexlerae* transplantation can alleviate cardiopulmonary symptoms induced by high-altitude exposure, protect the integrity of the intestinal barrier, and play a key role in promoting host adaptation to the hypobaric hypoxic conditions of high altitudes. Chen and colleagues reported that FMT from a plateau-specific species (plateau zokors) into low-altitude Sprague-Dawley rats effectively improved pulmonary metabolism, gene expression profiles, and respiratory function under hypoxic conditions, demonstrating significant potential of FMT for treating hypoxia-induced pulmonary hypertension (Chen et al., 2025).

Previous studies have demonstrated that acute cerebral ischemia can lead to gut microbiota dysbiosis, which in turn mediates neuroinflammatory responses through immune pathways (Benakis et al., 2016). FMT has been shown to reduce pro-inflammatory T cells in both the gut and brain, restore gut microbiota balance, improve stroke outcomes, and protect brain function after injury (Singh et al., 2016). Experimental results from Du et al. (2021) demonstrated that FMT could restore gut microbiota equilibrium, reduce serum and brain injury-side trimethylamine N-oxide levels, increase the expression of the antioxidant enzyme methionine sulfoxide reductase A in the hippocampus, decrease oxidative stress, mitigate neurological deficits following traumatic brain injury, and improve learning and spatial memory. Recent research further indicates that FMT, through modulation of the gut-brain-microbiota axis, increases the levels of growth hormone-releasing peptides in serum and cerebrospinal fluid, significantly reduces brain edema, promotes blood-brain barrier repair by inhibiting the TNF-α pathway, and maintains gut microbiota homeostasis while preserving neurological function (Zhang Y. et al., 2024). These findings suggest that FMT may offer novel therapeutic strategies for preventing and treating high-altitude sickness. Moreover, long-term or high-dose glucocorticoid therapy has been associated with disturbances in glucose and lipid metabolism. In patients with endogenous Cushing's syndrome, significant gut microbiota dysbiosis has been observed, characterized by an increased abundance of *Proteobacteria* and a decreased abundance of *Lachnospiraceae* and *Faecalibacterium* (Zhang et al., 2023c). Similar findings were reported by Zhang Q. et al. (2024) in a mouse model of dexamethasone-induced glucose and lipid metabolism disorders, which was also accompanied by a reduction in beneficial metabolites such as SCFAs. Notably, FMT was able to restore gut microbiota homeostasis and alleviate dexamethasone-induced metabolic dysfunction. This suggests that FMT may be a promising strategy for mitigating the side effects of dexamethasone in high-altitude regions and could potentially serve in the prevention and treatment of other high-altitude-related illnesses.

### 5.5 Pharmacologically active ingredients

Recent evidence suggests that quercetin may serve as a potential therapeutic agent targeting the gut-brain axis for the prevention of high-altitude sleep disorders. In a mouse model of high-altitude-induced sleep disturbances, oral administration of quercetin nanoparticles before ascent significantly prolonged sleep duration, improved hematological recovery, spontaneous behavior, and short-term memory, and reduced levels of inflammatory markers such as TNF-α and inducible nitric oxide synthase. These effects were likely associated with an increased abundance of *Lactobacillus* and *Lachnospiraceae* in the gut (Wu Y. et al., 2024). Additionally, citrus peel extract, which is rich in pectin and flavonoids, has been shown to effectively alleviate hypoxia-induced gut injury in mice and enhance gut integrity and barrier function by modulating the gut microbiota composition, particularly by increasing the abundance of the probiotic *Lactobacillus* (Yu et al., 2023). Furthermore, irbesartan has demonstrated efficacy in mitigating high-altitude hypoxia, pulmonary edema, and left ventricular systolic dysfunction caused by oxidative stress in rat models. This protective effect was attributed to an increased abundance of *Lactobacillaceae* and *Lachnospirillaceae* and a decreased abundance of *Prevotella* and *Desulfovibrionaceae* in the gut. The modulation of gut microbiota composition was associated with a reversal of elevated serum levels of angiotensin II, endothelin-1, IL-6, and C-reactive protein in HAPH model rats. Irbesartan also promoted increased activity of antioxidant enzymes such as superoxide dismutase and glutathione peroxidase, while reducing malondialdehyde levels in intestinal tissues (Nijiati et al., 2021).

Notably, traditional Chinese medicine (TCM) has demonstrated remarkable efficacy in preventing and treating high-altitude disorders (Wu Z. et al., 2024). Active components of commonly used medicinal herbs—including Panax ginseng, Eleutherococcus senticosus, and Rhodiola rosea—exhibit regulatory features paralleling those of gut microbiota modulation. Our team's prior research revealed that ginsenoside Rg3 alleviates hypoxia-induced inflammatory responses and oxidative stress at high altitudes, ameliorating altitude-related cardiac injury (Liu et al., 2025); Eleutheroside B restores cellular autophagy balance disrupted by high-altitude exposure through AMP-activated protein kinase/mammalian target of rapamycin signaling pathway activation, effectively mitigating HAPE (Pei et al., 2024). Concurrently, Jiang et al. (2022) demonstrated that salidroside from Rhodiola rosea not only reduces cerebral oxidative stress damage and inflammation induced by acute hypobaric hypoxia but also enhances energy metabolism via suppression of the nuclear factor kappa-B/nucleotide-binding oligomerization domain-like receptor protein 3 pathway. Furthermore, Titto et al. showed that curcumin strengthens alveolar epithelial barrier integrity and enhances alveolar fluid clearance in HAPE through anti-inflammatory effects and upregulation of tight junction proteins (Titto et al., 2020). Although direct evidence linking these effects to gut microbiota metrics remains limited, the overlapping mechanistic networks strongly suggest that TCM bioactive compounds may achieve their multi-target therapeutic effects through remodeling gut microbial ecosystems. Future investigations should employ metabolomic-microbiome integrative analyses to validate this hypothesis.

In summary, as synthesized in Table 3, a diverse arsenal of interventions—spanning oxygen enrichment, dietary modulation, probiotics, synbiotics, FMT, and pharmacological agents—demonstrates promising potential for preventing and treating high-altitude disorders by targeting the gut microbiome. However, these strategies are characterized by different levels of technological maturity and practical applicability. Supplemental oxygen and indigenous foods offer non-invasive and physiologically sound approaches, though their effects may be transient or context-dependent on dietary availability. Probiotic and synbiotic formulations present a more targeted strategy with a strong safety profile, yet their efficacy is often strain-specific and may not durably alter the entrenched gut ecosystem. Meanwhile, pharmacological agents like quercetin and citrus peel extracts provide a molecularly targeted alternative, leveraging defined compounds to modulate the microbiota; however, their efficacy can be constrained by bioavailability, potential off-target effects, and an incomplete understanding of their mechanism of action within the complex gut environment. In contrast, FMT represents the most potent approach for fundamentally restructuring gut microbiota, as evidenced by the causal role of Blautia wexlerae, but its complexity and the “black box” nature of donor material raise challenges for standardization and safety. Ultimately, the choice of intervention may be guided by the clinical scenario, balancing immediacy of effect against durability.

**Table 3:** Interventions targeting the gut microbiota for ameliorating high-altitude-related disorders.

**Intervention strategy**	**Affected key microbiota**	**Associated metabolites**	**Translational potential**	**Refs**
Oxygen enrichment	*Prevotellaceae* NK3B31 ↑*Ruminococcus* ↑	Butyrate ↑	Alleviate intestinal barrier damage.	Ma et al., 2023
Rubus chamaemorus, Rubus arcticus, Rubus ellipticus	–	Urolithin ↑	Anti-inflammatory, anti-oxidative stress, promoting high altitude health.	Burlando et al., 2023
*Brassica rapa* L. crude polysaccharides	*Lachnospiraceae bacterium* A2 ↓*Dorea* sp 52 ↓*Akkermansia muciniphila* ↑*Leuconostoc lactis* ↑*Enterococcus faecium* ↑	Butyrate ↑	Reduce hypoxic injury and inflammatory response, enhance intestinal barrier function.	Liu et al., 2024
Tibetan fermented milk	*Bacteroides* ↑*Faecalibacterium* ↑	–	Ameliorate cognitive dysfunction.	Liu J. et al., 2020
Resistant potato starch	*Bifidobacterium* ↑*Ruminococcus bromii* ↑*Clostridium chartatabidum* ↑	Butyrate ↑	Increase the abundance of probiotics and reduce disease susceptibility.	Baxter et al., 2019
*Lactobacillus delbrueckii subsp. bulgaricus* 4L3	*Prevotellaceae* UCG-001 ↓*Romboutsia* ↓	Linoleic acid ↑ Tyrosine ↑	Protect intestinal barrier functions, alleviate oxidative stress, and reduce inflammatory responses.	Song et al., 2024
Inulin	*Bifidobacterium* ↑*Lactobacillus* ↑	–	Improve intestinal morphology and increase food intake.	Ding et al., 2021
Synbiotics (constituting *Bifidobacterium bifidum, Bifidobacterium longum, Lactobacillus acidophilus, Lactobacillus rhamnosus, Saccharomyces boulardii, Streptococcus thermophilus*, and *Fructo oligosaccharides*)	*Prevotella* ↓*Paenibacillus* ↓*Clostridium* ↓*Turicibacter* ↓*Bacillus* ↓*Anoxybacillus* ↓*Enterococcus* ↓*Mucispirillum* ↓*Allobaculum* ↑*Lactococcus* ↑	–	Improve the morphology and function of the intestinal mucosal barrier and reduce inflammation.	Khanna et al., 2021
The probiotics supplement contains 3 *Bifidobacterium* strains (*B. animalis subsp. lactis* V9, *B. longum* KT-L9, *B. adolescentis* KT-A8) and 6 *Lactobacillus* strains (*L. casei Zhang, L. plantarum* P-8, *L. paracasei* KT-P6, *L. rhamnosus* M9, *L. acidophilus* KT-A1, *L. helveticus* H9); prebiotics consist of polydextrose, galactose, and inulin	*Ruminococcaceae* ↑*Lachnospiraceae* ↑*Lactococcus* ↑*Parabacteroides* ↑*Alistipes* ↑*Prevotella* ↓	–	Attenuate cardiac hypertrophy inflicted by hypobaric hypoxia exposure.	Hu et al., 2022
*Bacillus subtilis* BS1 and BS2, *Bacillus velezensis* BV1	–	–	Improve antioxidant capacity, anti-inflammatory capacity, and immune function.	Li et al., 2019
*Blautia wexlerae* DSM19850	*Ligilactobacillus* ↑	–	Alleviate high-altitude related cardiopulmonary injury.	Su et al., 2024b
FMT (from plateau zokors to low-altitude Sprague-Dawley rats for 30 days)	*Prevotellaceae* UCG-001 ↑*Prevotellaceae* NK3B31 ↑*Lachnospiraceae* NK4A136 ↑	–	Relieve hypoxic pulmonary hypertension.	Chen et al., 2025
Oral quercetin nanoparticles	*Lactobacillus* and *Lachnospira* ↑	–	Alleviate high-altitude sleep disturbance.	Wu Y. et al., 2024
Citrus tangerine pith extract	*Lactobacillus* ↑	–	Enhance intestinal integrity and barrier function.	Yu et al., 2023
Irbesartan	*Lactobacillaceae* and *Lachnospiraceae* ↑*Prevotellaceae* and *Desulfovibrionaceae* ↓	–	Ameliorate high altitude pulmonary hypertension.	Nijiati et al., 2021

This table systematically categorizes strategies aimed at modulating the gut microbiota to prevent or treat high-altitude illnesses. It lists the intervention strategy, affected key microbiota (specifying species/strain where available, with ↑/↓ denoting enrichment or reduction), associated metabolites, translational potential, and supporting references. FMT, fecal microbiota transplantation; *B. animalis subsp. lactis* V9, *Bifidobacterium animalis subsp. lactis* V9; *B. longum* KT-L9, *Bifidobacterium longum* KT-L9; *B. adolescentis* KT-A8, *Bifidobacterium adolescentis* KT-A8; *L. casei* Zhang, *Lactobacillus casei* Zhang; *L. plantarum* P-8, *Lactobacillus plantarum* P-8; *L. paracasei* KT-P6, *Lactobacillus paracasei* KT-P6; *L. rhamnosus* M9, *Lactobacillus rhamnosus* M9; *L. acidophilus* KT-A1, *Lactobacillus acidophilus* KT-A1; *L. helveticus* H9, *Lactobacillus helveticus* H9; Refs, References.

## 6 Impact of gut microbiota on drug metabolism in high-altitude environments

At sea level, the influence of gut microbiota on pharmacokinetics has been extensively studied (Cai et al., 2023). Equally noteworthy is that hypobaric hypoxia at high altitudes induces gut microbial restructuring (Bai et al., 2022a), leading to significant alterations in pharmacokinetic properties that subsequently affect the therapeutic efficacy and safety of orally administered drugs (Zhou et al., 2018). Experimental studies demonstrate that acute high-altitude exposure alters gut microbial composition in rats, resulting in a 41.29% reduction in clopidogrel's area under the plasma concentration-time curve (AUC) and a 36.30% decrease in peak concentration (Cmax), with plasma clearance (CL) increasing 2.3-fold (Zhang et al., 2023). Enhanced metabolism and reduced absorption of clopidogrel may diminish its bioavailability. Parallel research reports indicate that high-altitude-induced gut microbiota changes in rats elevate nifedipine's AUC by 39.10%, while the time to peak drug concentration and CL decrease by 48.91% and 34.71%, respectively (Zhang et al., 2018a). Elevated plasma nifedipine levels may increase the frequency of dose-dependent adverse effects in high-altitude settings. Sun et al. (2020) experimentally demonstrated that reduced abundances of *Prevotella copri* and *Enterococcus faecalis* in acute high-altitude-exposed rat models correlate with inhibited aspirin metabolic activity, manifested by 82.20% and 61.03% increases in AUC and Cmax, alongside 43.55% and 31.73% reductions in CL and thromboxane B2 levels. Consequently, dosage reduction of aspirin is clinically warranted upon high-altitude exposure to mitigate bleeding risks and gastrointestinal complications.

Mechanistically, gut microbiota modulates drug metabolism through two primary pathways: direct participation via microbial enzyme synthesis and metabolite production, and indirect regulation by altering drug-metabolizing enzymes [e.g., cytochrome P450 (CYP450)] and drug transporters, thereby modifying drug bioavailability, bioactivity, or toxicity (Qiu et al., 2024). β-Glucuronidase, a representative microbial enzyme secreted by gut bacteria, catalyzes the hydrolysis of glucuronide conjugates and xenobiotic detoxification processes. β-Glucuronidase critically influences the hepatotoxicity of tacrine (Yip et al., 2018) and the gastrointestinal toxicity of irinotecan and non-steroidal anti-inflammatory drugs (Pellock and Redinbo, 2017; Stringer et al., 2008). Gut microbial-derived carboxylesterases and azoreductases promote the metabolism of angiotensin-converting enzyme inhibitors and sulfonamide drugs, respectively (Sun et al., 2019; Zhang et al., 2018b). Beyond enzymatic activity, microbiota-generated metabolites—including SCFAs, phenolic compounds, and bile acids—participate in pharmacological regulation. For instance, *Akkermansia muciniphila* alleviates acetaminophen-induced liver injury by increasing SCFAs secretion to mitigate oxidative stress and inflammation (Xia et al., 2022). Conversely, *Clostridioides difficile*-produced p-cresol competes with acetaminophen for sulfotransferase binding, impairing acetaminophen bioavailability (Clayton et al., 2009). Similarly, bile acids compete with statins for the solute carrier organic anion transporter family member 1B1 during enterohepatic circulation, altering statin pharmacokinetics (Kaddurah-Daouk et al., 2011). Furthermore, gut microbial metabolites regulate drug metabolism by modulating the expression and activity of nuclear receptors such as the pregnane X receptor, constitutive androstane receptor, and aryl hydrocarbon receptor. Experimental evidence demonstrates that microbiota-derived tryptophan metabolites act as pregnane X receptor agonists, inducing CYP3A4 and multidrug resistance protein 1 (MDR1) expression (Illes et al., 2020). In contrast, tryptophan catabolites antagonize aryl hydrocarbon receptor activation and suppress CYP1A2 expression (Morgan et al., 2018).

The emerging field of high-altitude pharmacomicrobiomics has revealed the gut microbiota's role as a master regulator of xenobiotic metabolism under hypoxic stress (Qiu et al., 2024), though mechanistic evidence remains preliminary. Bai et al. (2022b) demonstrated that hypoxia in the Qinghai-Tibet Plateau downregulates CYP3A1 and MDR1 expression at both protein and mRNA levels in rats, potentially linked to reduced aerobic taxa (e.g., *Lactobacillus rhamnosus*) and enriched anaerobes (e.g., *Clostridium*) in the gut microbiome. Notably, diminished *Lactobacillus rhamnosus* and *Clostridium* abundances may drive MDR1 suppression, potentially mediated via microbial extracellular vesicles. Furthermore, CYP3A1 emerges as one of the most significantly downregulated CYP450 isoforms under high-altitude exposure, suggesting gut microbiota as a key mediator of CYP3A1 modulation—a plausible mechanism for hypoxia-driven microbial regulation of drug metabolism. Crucially, bidirectional microbiota-drug enzyme/transporter interplay may exist, necessitating further mechanistic interrogation.

Published studies position the gut microbiome as a hypoxia-responsive “second liver” with altitude-specific metabolic signatures. However, current evidence predominantly derives from animal models and *in vitro* systems, underscoring the need for large-scale, multicenter clinical trials in high-altitude populations to validate drug-microbiome interactions. Longitudinal multiethnic cohort studies tracking dynamic microbiota-drug metabolism alterations across altitude acclimatization phases could enable microbiome-informed dosing algorithms, critically advancing pharmacological safety and personalized therapeutics in high-altitude medicine.

## 7 Gut microbiota biomarkers: challenges and opportunities in high-altitude medicine

The dynamic plasticity of the gut microbiota offers a unique opportunity to identify biomarkers predictive of high-altitude pathologies, although current evidence remains preliminary. Through a meta-analysis of populations on the Qinghai-Tibet Plateau in China, Zhao et al. (2024) identified universally applicable gut microbiota biomarkers and their association with high-altitude adaptation. Their study found that depletion of butyrate-producing bacteria (such as Ruminococcaceae and Lachnospiraceae) correlated with the severity of polycythemia in CMS; mechanistic validation demonstrated that microbially derived butyrate alleviates hypoxic intestinal injury by inhibiting lactate accumulation and overactivation of HIF-1α (Zhao et al., 2024). Despite this breakthrough, the cross-sectional nature of such studies limits causal inference and validation of biomarker specificity. Prospective cohort studies tracking microbial dynamics from pre-altitude exposure baselines to disease onset are crucial for establishing their predictive value. Encouragingly, the latest research reports indicate that reduced abundances of *Veillonella rogosae* and *Streptococcus rubneri*, alongside decreased levels of their associated metabolites (including L-aspartic acid, α-ketoglutarate, and betaine), significantly impact energy metabolism processes such as glycolysis. This may exacerbate myocardial maladaptation to hypobaric hypoxia, thereby triggering high-altitude cardiac damage (Zhou et al., 2025). *Veillonella rogosae* and *Streptococcus rubneri*, and their associated serum metabolites, can serve as microbiome and metabolome biomarkers, respectively (Zhou et al., 2025), providing novel avenues for the early diagnosis and potential therapeutic intervention of high-altitude heart disease.

Translating microbial biomarkers into clinical tools faces two fundamental challenges. First, overlapping microbial alterations in CMS and non-altitude disorders (e.g., inflammatory bowel disease)—such as *Enterobacteriaceae* enrichment (Huttenhower et al., 2014)—necessitate integrative approaches combining taxa profiling, metabolite gradients (e.g., inosine/uric acid ratios), and host genetics to enhance biomarker specificity. Second, current temporal resolution inadequately captures pre-clinical microbial transitions. Innovations like wearable biosensors for continuous gut microbiome monitoring (Ngashangva and Chattopadhyay, 2023), coupled with longitudinal metagenomic profiling, could revolutionize real-time tracking of host-microbe interactions during altitude adaptation. Furthermore, FMT from extreme adapters (e.g., Sherpas; Gilbert-Kawai et al., 2014) into hypoxic chamber-exposed germ-free models may help isolate causal biomarkers. The diagnostic and therapeutic utility of gut microbial biomarkers in high-altitude medicine will ultimately depend on the multidisciplinary integration of these advanced technologies with mechanistic validation, enabling translation beyond associative observations into actionable clinical insights.

## 8 Synthesis and perspectives

### 8.1 Conclusion

High-altitude exposure, characterized by hypoxia and accompanying stressors, poses a significant threat to physiological health. In this context, the gut microbiota serves as a pivotal interface between environmental pressure and host physiology, playing a central role in the adaptation process. This review synthesizes current evidence demonstrating that the gut microbiota undergoes substantial remodeling under high-altitude conditions. The resultant microbial community orchestrates systemic adaptation by enhancing energy harvest and metabolic efficiency. This metabolic shift is primarily driven by the enrichment of taxa that enhance the production of SCFAs and other key energy substrates. Furthermore, the restructured microbiota promotes host health by modulating immune responses, strengthening intestinal barrier integrity, and mitigating oxidative stress. It also influences critical neuroendocrine pathways through microbial neurotransmitters and metabolites, thereby helping to regulate systemic functions such as mood and blood pressure under hypoxic stress. However, when the host's adaptive capacity is exceeded, this delicate equilibrium is disrupted, and the altered microbial community may transition to a pathogenic state, thereby contributing to the development of various high-altitude-related diseases. This mechanistic insight establishes the gut ecosystem as a promising therapeutic target. Consequently, interventions aimed at modulating the gut microbiota—including oxygen enrichment, indigenous foods, probiotic and/or prebiotic supplementation, FMT, and treatment with pharmacologically active components—hold significant potential for preventing and managing high-altitude maladies. The continued translation of gut microbial biomarkers and targeted interventions into clinical practice is expected to advance the field of high-altitude medicine and improve human resilience in these extreme environments.

### 8.2 Limitations and future directions

Although this review synthesizes substantial evidence linking the gut microbiota to high-altitude adaptation and pathology, several limitations within the current literature merit attention. A primary challenge lies in the pronounced heterogeneity across studies. Direct comparisons and meta-analyses are hindered by inconsistencies in altitude definitions, cohort types—including indigenous populations, sojourners, and athletes—and exposure duration. Additionally, influential confounding factors such as dietary habits, host genetics, and complex environmental variables are often insufficiently controlled, complicating the isolation of hypoxia's independent effects on the gut microbial ecosystem. Although studies have compellingly documented altitude-associated microbial changes, most have failed to establish definitive causality. It remains uncertain whether these microbial shifts are drivers of physiological adaptation or merely secondary responses to environmental stress. Furthermore, the field is predominantly characterized by correlative observations derived mainly from 16S rRNA gene sequencing, which limits taxonomic resolution predominantly to the genus level and creates a gap for clinical translation.

Future research needs to transcend current limitations to advance mechanistic insight and clinical translation in this field. Several strategic directions appear particularly promising. First, controlled-environment studies using hypobaric chambers or environmental cabins would help standardize key variables such as diet, temperature, and light cycles (Wagner and Kleiner, 2025). This integrated approach enables precise manipulation of oxygen levels while isolating hypoxic effects from other confounding factors. Second, future investigations would benefit from adopting deeply phenotyped, multi-omics longitudinal cohorts that prospectively collect high-resolution metadata on host genetics, diet, and physical activity (Consortium, 2019; Reicher et al., 2025). The resulting rich datasets would enable the application of multivariate statistical models to partition the influence of confounding variables. Concurrently, emerging computational tools such as METALICA (Ruiz-Perez et al., 2024) could be applied to infer microbial gene regulatory networks and quantify trait heritability from metagenomic data, thereby elucidating the interplay between host genetics and environmental exposure. Furthermore, moving beyond 16S rRNA sequencing to integrated multi-omics frameworks (particularly metagenomics and metabolomics) will be crucial for identifying key bacterial strains and effector metabolites. Finally, microbiota-targeted interventions stemming from these mechanistic insights should be validated through large-scale, randomized controlled trials in high-altitude populations to ensure successful clinical translation.
